# Effect of Methylmercury Binding on the Peroxide-Reducing Potential of Cysteine and Selenocysteine

**DOI:** 10.1021/acs.inorgchem.0c03619

**Published:** 2021-02-15

**Authors:** Andrea Madabeni, Pablo A. Nogara, Marco Bortoli, João B. T. Rocha, Laura Orian

**Affiliations:** † Dipartimento di Scienze Chimiche, Università degli Studi di Padova, Via Marzolo 1, 35131 Padova, Italy; ‡ Departamento de Bioquímica e Biologia Molecular, Universidade Federal de Santa Maria (UFSM), 97105-900 Santa Maria, RS, Brazil

## Abstract

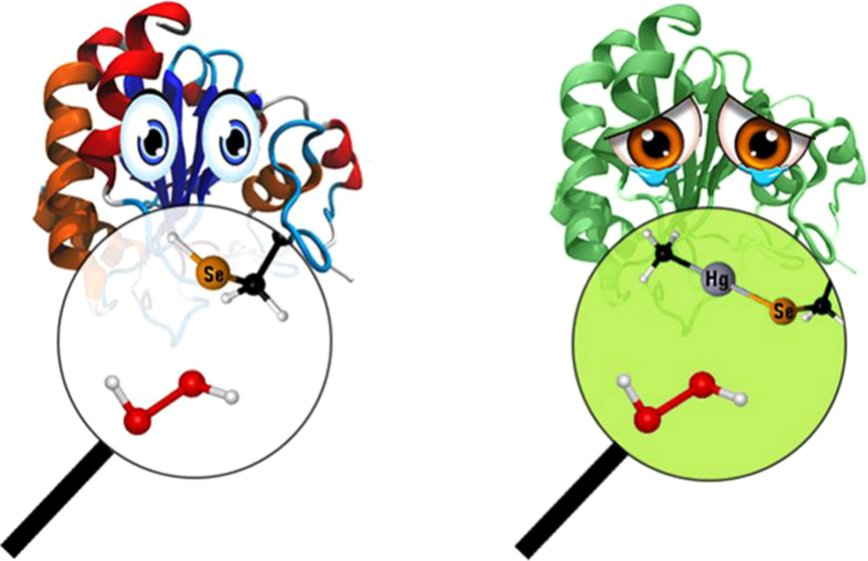

Methylmercury (CH_3_Hg^+^) binding to catalytically fundamental cysteine and selenocysteine of peroxide-reducing enzymes has long been postulated as the origin of its toxicological activity. Only very recently, CH_3_Hg^+^ binding to the selenocysteine of thioredoxin reductase has been directly observed [PickeringI. J.Inorg. Chem., 2020, 59, 2711−27183204951110.1021/acs.inorgchem.9b03072], but the precise influence of the toxicant on the peroxide-reducing potential of such a residue has never been investigated. In this work, we employ state-of-the-art density functional theory calculations to study the reactivity of molecular models of the free and toxified enzymes. Trends in activation energies are discussed with attention to the biological consequences and are rationalized within the chemically intuitive framework provided by the activation strain model. With respect to the free, protonated amino acids, CH_3_Hg^+^ binding promotes oxidation of the S or Se nucleus, suggesting that chalcogenoxide formation might occur in the toxified enzyme, even if the actual rate of peroxide reduction is almost certainly lowered as suggested by comparison with fully deprotonated amino acids models.

## Abstract 2

The TOC shows a close-up on the catalytic pocket of a healthy (left) and sick (right) glutathione peroxidase. On the left, a selenocysteine can be seen interacting with hydrogen peroxide, while on the right, the relative methylmercury complex alters the peroxide-reducing potential of the chalcogen nucleus. These two situations have been investigated in this work.

## 1 Introduction

Physiological thiols and selenols are widely recognized as methylmercury (CH_3_Hg^+^) targets.^1−4^ In the biological environment, cysteine (Cys) and selenocysteine (Sec) constitute the main thiol- and selenol-containing compounds and are catalytically fundamental residues in the enzymatic activity of the glutathione peroxidase (GPx) and thioredoxin reductase (TrxR) families, whose inhibition has been demonstrated to be implicated in CH_3_Hg^+^ toxicity.^2,5−7^ These enzymes, particularly GPxs, are peroxide-reducing enzymes, which contribute to keep regulated the peroxide tone of the cell.^8^ CH_3_Hg^+^ possesses pro-oxidative properties, likely via GPx inhibition, which leads to the accumulation of hydroperoxide and to hydroperoxide-mediated excitotoxicity, which have been associated with its neurotoxicity.^4,6,9^ Despite the toxicological knowledge about GPx inhibition by CH_3_Hg^+^, the chemical details behind its interaction with thio- and selenoproteins are not known, and precise knowledge about its toxicological mechanism has not been achieved.^3^ Particularly, only very recently, the binding between the toxicant and the catalytically fundamental Sec of TrxR has been observed directly,^10^ in an elegant study by Pickering et al. They succeeded in detecting the Se–Hg bond by means of extended X-ray absorption fine structure spectroscopy.

The effect of CH_3_Hg^+^ binding on the chalcogen nucleus implicated in the catalytic mechanism of such peroxide-reducing enzymes has never been investigated. In fact, sulfur and selenium possess a central role in the antioxidant system of living beings and both endogenous antioxidant molecules and peroxide-reducing enzymes employ S or Se to fulfill their role. Seminal computational investigations on (methyl)mercury chalcogenolate complexes have been previously performed by Schreckenbach et al.^11^ Particularly, they highlighted how the chalcogenophilicity of mercury is the same in systems of increasing complexity,^12^ and they investigated peculiar reactivity aspects of free methylmercury selenocysteinate complexes, which leads to their degradation.^13^ In fact, methylmercury–selenium binding is on the basis of the so-called selenium–mercury antagonism. Hg-containing compounds can cause selenium depletion due to the formation of mercury selenide nanoparticles (HgSe), which could disrupt the synthesis of selenoenzymes and increase Hg neurotoxicity. In contrast, HgSe formation could also antagonize CH_3_Hg^+^ toxicity because of the far less toxic properties of such nanoparticles.^14−16^ However, a careful investigation of how CH_3_Hg^+^ affects the peroxide-reducing capabilities of Cys and Sec residues is still missing and it is important to understand the fate of the enzyme after CH_3_Hg^+^ binding.

**Scheme 1:**
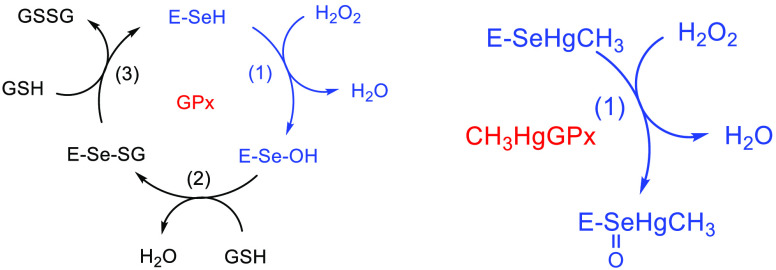
GPx Catalytic Cycle (Left) The first step (1) Is the actual peroxide reduction, displaying the conversion of a selenol (−SeH) to a selenenic acid (−SeOH) that can be reduced back to a selenol by two glutathione (GSH) molecules. (Right) CH_3_Hg^+^-inhibited Sec GPx mechanism of peroxide reduction, as has been postulated. The step, equivalent to step (1) of the functional GPx, displays the oxidation of a methylmercury selenolate to a selenoxide, with consequent peroxide reduction.

It is well known that GPx operates via a three-step mechanism (Scheme 1, left), where the first step is the effective peroxide reduction.^17,18^ Since the first step can in principle occur with some changes even after CH_3_Hg^+^ binding (Scheme 1, right), such an event deserves deeper scrutiny, and it is the main topic of this work.

The GPx first step has recently attracted great attention, and it has been demonstrated that for GPx^18,19^ and other important Cys/Sec-based enzymes,^20^ peculiar features of the catalytic pocket enable Cys and Sec to display peroxidatic activity. Particularly, Cys and Sec can become deprotonated through a proton transfer to a nearby acceptor site, leading to a charge-separated intermediate. When (Cys/Sec)^−^ attacks one oxygen of the peroxide bond, the proton is shuttled to the opposite oxygen atom, enabling an efficient cleavage of water from the substrate. Such a mechanism, however, is only possible in suitably designed molecular architectures like a catalytic pocket and requires a proton-acceptor in a specific position, optimal for both deprotonating Cys/Sec and donating the proton to the peroxide bond. Evidently, after complexation by CH_3_Hg^+^, the formation of a charge-separated intermediate is inhibited, and this might be enough to impair enzyme functionality. However, a thorough investigation of the effect of CH_3_Hg^+^ binding to thio- and selenoproteins based on molecular models is recommended.

*In silico* investigation on the oxidation of molecular organochalcogen compounds is not unusual in the literature,^21−23^ and for both organosulfur and organoselenium compounds, different mechanistic pathways have been investigated.^24−27^ Particularly, focusing on thiols, it has been proven that the reaction occurs faster when the system is deprotonated or when deprotonation occurs at the transition state, in a proton shuttling manner.^26^ However, focusing on Cys, Sec, and tellurocysteine (Tec), the comparative studies are rare. Particularly, Cardy et al.^27^ compared Cys to Sec oxidation by hydrogen peroxide (H_2_O_2_) in their deprotonated form, noting an important impact of the conformation on the activation energy, with Sec having a moderately lower barrier with respect to Cys of about 3 kcal mol^–1^. Thus, they concluded that the Sec peroxidatic behavior is boosted only inside the catalytic pocket of GPxs. In 2017, Bortoli et al. showed that in a cluster model of GPx, a charge separation pathway is still available also for Cys instead of Sec, while such a mechanism was not identified for Tec, likely because of the hydride character of the Te–H bond.^28^

The scope of this work is to understand the influence of CH_3_Hg^+^ on the chalcogen nucleus oxidation by hydrogen peroxide in model methylmercury (seleno/telluro) cysteinate complexes. While (seleno)cysteine binding with CH_3_Hg^+^ is relevant for toxicological reasons, tellurocysteine is included for completeness and because tellurols might have a role in methylmercury detoxification in virtue of their high mercury binding capabilities.^29,30^

## 2 Computational Methods

All DFT calculations have been performed with the Amsterdam Density Functional (ADF) program^31−33^ 2018 and 2019 version. Zeroth-order regular approximation (ZORA) has been employed to include relativistic effects in the calculations, as recommended in the presence of heavy atoms.^34^ In all calculations, BLYP^35,36^ functional has been used with the inclusion of Grimme dispersion with the Becke–Johnson damping function.^37−40^ For all atoms, the TZ2P basis set has been used, which is a large uncontracted set of Slater-type orbitals of triple-ζ quality, augmented with two sets of polarization functions per atom. In all calculations, small frozen core approximation has been employed. Such a level of theory is from now on denoted as ZORA–BLYP-D3(BJ)/TZ2P and was previously benchmarked for methylmercury chalcogenolate structures and reactivity.^30^ Moreover, it has been previously applied for the investigation of the oxidation of organochalcogen compounds by H_2_O_2_.^41^^41^ For solvent-assisted proton-exchange (SAPE) calculations, activation energies have been computed also employing B3LYP^36,42,43^ functional on the BLYP-D3(BJ) optimized geometries, since hybrid functionals with a low percentage of Hartree–Fock exchange are recommended to quantitatively model proton transfer reactions.^44,45^ The trends obtained are consistent with those obtained employing BLYP-D3(BJ) functional and are thus not discussed in the main text (Table S2). For all fully optimized structures, frequency calculations have been performed to assess whether or not a true minimum was reached. All minima have real frequencies, while transition states have one imaginary frequency associated with the normal mode connecting reactants to products. Intrinsic reaction coordinate (IRC) calculations have been performed for a representative set of reactions to obtain the minimum-energy path connecting the transition state to the two closest minima (reactant or reactant complex and product or product complex).^46^ For IRC calculations, the 2019 version of ADF has been fundamental to reach geometry convergence when methylmercury was involved. Solvation effects (water) have been taken into account by means of the conductor-like screening model (COSMO).^47,48^ The relative dielectric constant used for water is 78.39, while the empirical parameter in the scaling function of the COSMO equation has been chosen as 0.00. MM3 radii^49^ divided by 1.2 have been used as by default in ADF when performing COSMO calculations. For the solvent-excluding surface of water, we used an effective radius of 1.93 Å, derived from the macroscopic density. All of the calculations have been performed in gas phase, and subsequent single-point calculations in solvent (water) for the biologically relevant reactions involving Cys and Sec show no change in activation or reaction energies trends (Tables S3 and S4). Molecular structures have been illustrated using CYLview.^50^

To gain qualitative and quantitative insights into the CH_3_Hg^+^ effect, activation strain analysis (ASA) and energy decomposition analysis (EDA) have been performed along the whole reaction coordinate (rc) or at the reactant complex and transition state only.^51−54^ ASA is a fragment-based approach that allows decomposition of the energy of any point along the rc into two contributions
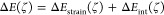
1where Δ*E*_strain_(ζ) is the deformation energy required to distort the reactants into the geometry they display at the point ζ along the rc, while Δ*E*_int_(ζ) accounts for the chemical interactions between the distorted reactants.

**Scheme 2:**
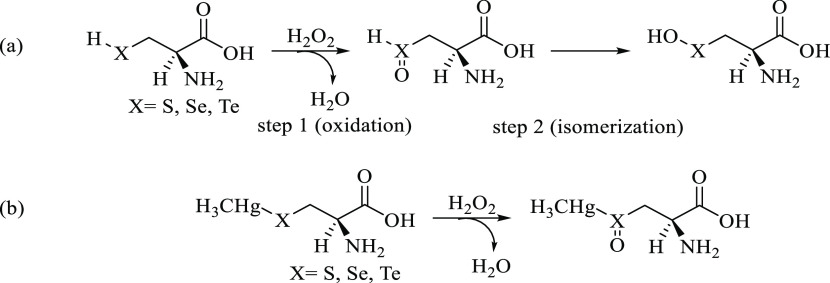
(a) Oxidation of Cys, Sec, and Tec to Chalcogenoxide Followed by Isomerization to Chalcogenenic Acid; (b) Oxidation of MeHgCys, MeHgSec, and MeHgTec to Chalcogenoxide

Δ*E*_int_(ζ) can be further decomposed into different chemically meaningful terms within the EDA scheme
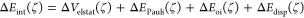
2where Δ*E*_elstat_(ζ) accounts for the semiclassical electrostatic interaction between the unperturbed electronic densities of the two approaching fragments; Δ*E*_Pauli_(ζ), namely, Pauli repulsion, the repulsive interaction between occupied orbitals, and Δ*E*_oi_(ζ), the orbital interaction [such as highest-occupied molecular orbital (HOMO)–lowest-unoccupied molecular orbital (LUMO) interaction] and the dispersive interaction Δ*E*_disp_(ζ), which is taken into account at the level of theory of the calculations [i.e., D3(BJ)]. ASA and EDA at single-point geometries have been executed manually as implemented in ADF. Conversely, along the whole rc, they have been performed using IRC geometries with the program PyFrag.^55^

Gibbs free energies at 298.15 K and 1 atm have been computed by means of standard statistical-thermodynamics relationships within the ideal gas approximation, employing electronic energies and frequencies. Activation and reaction free energies display the same trends (Tables S5 and S6) and are not discussed in the main text to keep consistency with ASA and EDA that can be performed on electronic energies only.

## 3 Results and Discussion

To gain insight into how CH_3_Hg^+^ binding affects the peroxide-reducing potential of Cys, Sec, and Tec, we have investigated *in silico* the mechanistic details of Cys, Sec, and Tec oxidation by H_2_O_2_ and the analogous mechanisms for methylmercury cysteinate, selenocysteinate, and tellurocysteinate complexes (MeHgCys, MeHgSec, and MeHgTec, respectively). For completeness, reaction and activation energies have been calculated also for Cys^–^ and Sec^–^, which are the two relevant systems from a biochemical point of view of catalysis, to take into account the effect of full deprotonation as it might occur in solution at medium–high pH, and in the active site of peroxidatic enzymes. These systems have been chosen as models of the fully functional and toxified enzymes. However, even such situation does not exactly match to the one occurring inside GPx, where the chalcogenolate attack to H_2_O_2_ occurs without any appreciable barrier and thus is not rate-determining.^18^

Three different mechanisms have been investigated for the amino acid oxidation. First, a stepwise mechanism has been followed for the oxidation of the chalcogenols to the corresponding chalcogenenic acids, via a chalcogenoxide intermediate (Scheme 2a).

Such a mechanism displays high activation energies and significantly differs from the enzymatic one, especially because of the high isomerization barrier required to pass from the chalcogenoxide to the chalcogenenic acid.^24,26^ Conversely, the oxidation to telluroxide has been hypothesized for Tec in mechanistic studies in enzyme, and it is likely possible also for the free Tec.^28^ Even if this pathway is unlikely to be responsible for Cys/Sec oxidation to the relative acids, it is valuable from a theoretical point of view: in fact, replacing −H with −HgCH_3_, the oxidation to chalcogenoxide can be modeled in an identical way, thus showing us the mere effect of the substituent (Scheme 2b).

**Scheme 3:**
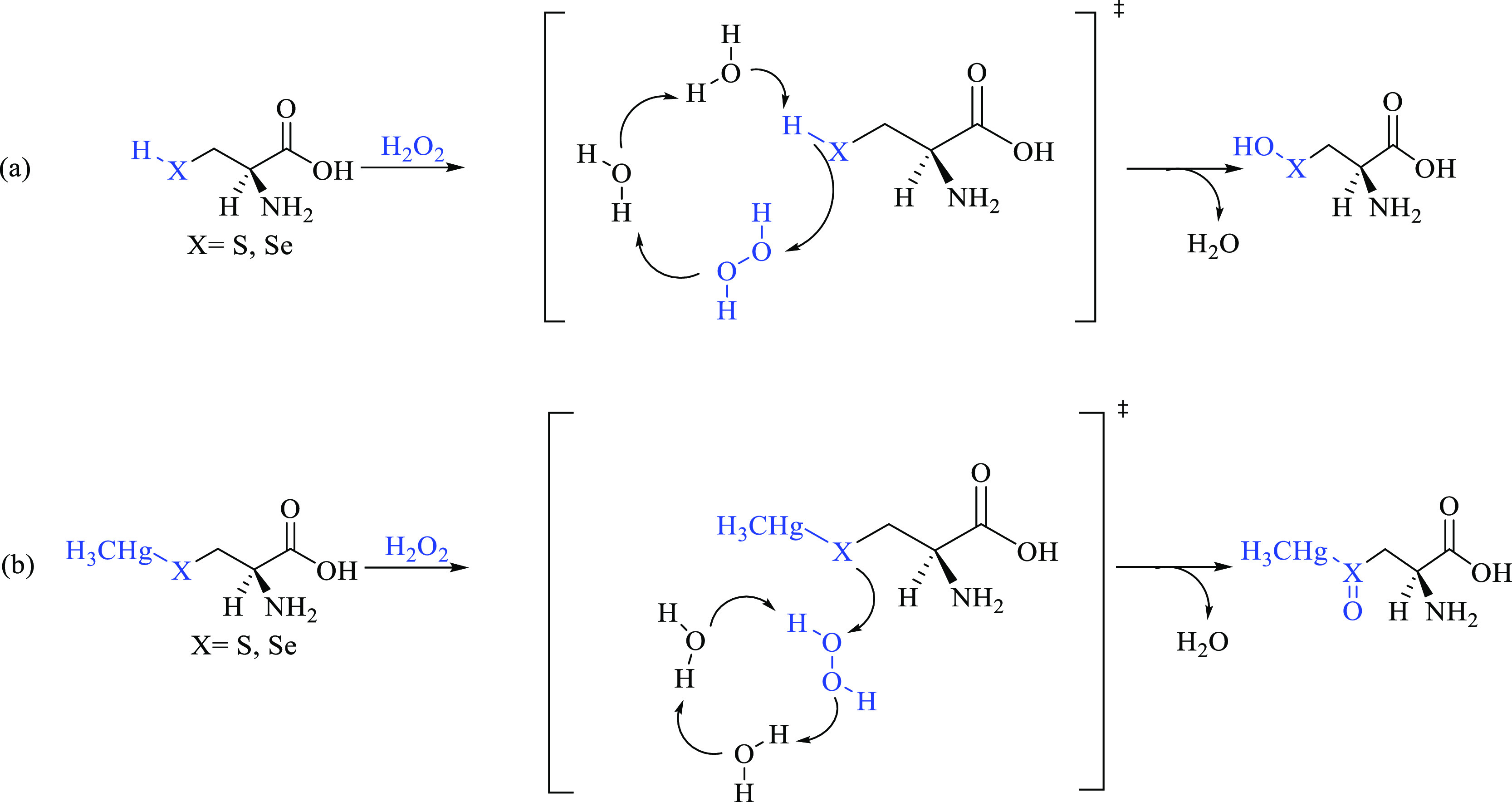
(a) Cys and Sec Direct Oxidation to Chalcogenenic Acid and (b) MeHgCys and MeHgSec Oxidation to Chalcogenoxide via Solvent-Assisted Proton Exchange The proton shuttling at the transition state is depicted in parentheses.

To more properly model the oxidation of our systems in the presence of a few water molecules (as they are usually present in the catalytic pockets of enzymes such as GPx), a second pathway has been investigated employing the so-called solvent-assisted proton-exchange (SAPE) approach, which has been extensively applied by Bayse et al. to organochalcogen reactivity.^22,25,56^ In this mechanism, a proton shuttles through a network of hydrogen-bonded water molecules and H_2_O_2_ and leads to peroxide reduction in a concerted manner. We employed two water molecules in our SAPE network (Scheme 3), because only a couple of them are usually present inside the catalytic pocket of GPx.

**Scheme 4:**
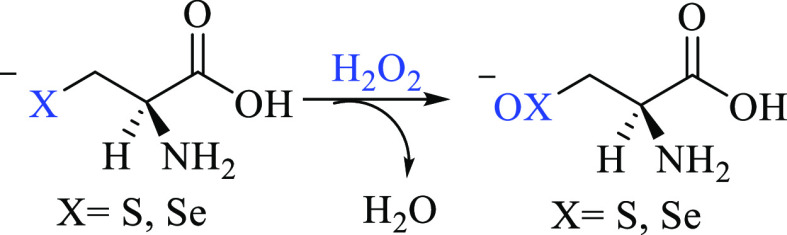
Cys^–^ and Sec^–^ Direct Oxidation to Deprotonated Chalcogenenic Acids

Finally, the direct oxidation of Cys^–^ and Sec^–^ has been followed along a concerted mechanism analogous to that previously reported for these amino acids^27^ and for simpler chalcogenolates.^24,57^ This pathway, which leads to water and to deprotonated chalcogenenic acids, will be called anionic mechanism (Scheme 4).

The GPx-like, stepwise mechanism via a charge-separated intermediate has not been investigated because it would have required the definition of an enzyme-specific proton-acceptor as it has been recently done in a work by some of us, and it was shown to display unfeasible activation energies when the whole enzyme environment is not involved.^58^ It is possible, however, that the introduction of a proton acceptor strategically placed near the substrate/hydrogen peroxide might promote a proton shuttling mechanism, thus further lowering the activation energy of the SAPE mechanism.

Cys, Sec, and Tec have been optimized starting from the minimum-energy conformer reported in the literature for Cys.^59^ For the relative complexes with methylmercury, −H has been replaced with −HgCH_3_ and a full optimization was carried out, while for the anionic structures, we reoptimized after H^+^ removal. The oxidation to chalcogenoxide can proceed along two diasteroisomeric pathways, due to Cys/Sec/Tec stereogenic carbon atom and to the chalcogen nucleus at which the oxidation occurs. No great energetic or mechanistic differences have emerged along the two pathways for organoseleno oxidation.^60^ Thus, only one pathway has been fully investigated. With the aim of understanding the CH_3_Hg^+^ effect, we have followed the path along which the reaction energies with and without CH_3_Hg^+^ showed slightly greater differences. Thus, we have not followed the path going through the lowest-energy diastereoisomer, but rather the one through the least stable as it followed from preliminary energetic analysis on methylselenocysteine (MeSec) and methylmercury selenocysteinate (MeHgSec). For the protonated systems and methylmercury complexes, we followed the oxidation of the (R, R) diastereoisomer, for which both the stereogenic carbon of the amino acids and the stereogenic chalcogen atom have the R absolute configuration. For the anionic mechanism, we ensured to follow the reaction pathway leading to structurally analogous products.

### 3.1 Mechanistic Details

**Figure 1:**
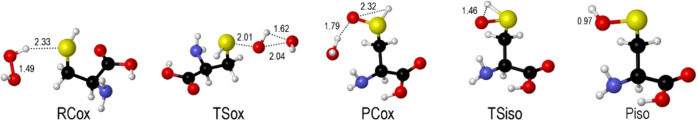
Relevant stationary points along the stepwise oxidation mechanism for Cys. Relevant interatomic distances are given in Å. Analogous structures were optimized for Sec and Tec.

The stepwise pathway (Scheme 2) has been found for all three residues, and the oxidative step (Scheme 2, step 1) has been found also when replacing −H with −HgCH_3_ (Scheme 2b). In all cases, the reaction goes through similar structures. First, a reactant complex (RCox) is formed, where hydrogen peroxide is located in proximity of the chalcogen nucleus. The oxidation takes place crossing a transition state (TSox), where the O–O bond of hydrogen peroxide is breaking apart, while the O–X (X = S, Se, Te) bond is forming. The reaction leads to a weakly bonded product complex (PCox) with a water molecule coordinated to the chalcogenoxide group. The oxidized product (Pox) is the same species after removal of the water molecule (i.e., the water molecule and the chalcogenoxides are infinitely distant and are thus considered free products). Upon oxidation, the conformation of the amino acids slightly changes, due to the interaction between the aminic function and the chalcogenoxide moiety, as previously observed for methylselenocysteine.^61^ Analogous structures were located for the corresponding methylmercury complexes, with a few minor differences: in the reactant complexes, H_2_O_2_ is also in proximity of Hg nucleus, and in the oxidized products, the slightly rotated conformation has not been located because the complex retains the same conformation of the reactant after oxidation. For the free amino acids, the isomerization occurs crossing a transition state (TSiso) that connects the chalcogenoxide Pox to the chalcogenenic acid Piso (Figure 1). For the complexes, the isomerization pathway to the chalcogenenic acid is not possible; thus, only the oxidation to chalcogenoxide has been investigated, even if further evolution of the chalcogenoxide cannot be ruled out. The oxidative step of the stepwise mechanism for free amino acids and the oxidation to chalcogenoxide for the complexes will be from now on referred to as the minimal mechanism.

All of the reactions are characterized by a high-energy transition state for the isomerization process, as previously reported (Figure S1), with the activation energy required for the oxidative step that decreases when going from Cys to Sec and to Tec, in agreement with the enhanced peroxidatic activity of organoseleno and organotelluro compounds with respect to the analogous organosulfur compounds (Table 1).^24,41^

**Table 1:** Electronic Energies (kcal mol^–1^) Relative to Free Reactants for the Oxidation (Minimal and Anionic Mechanism) of Free Protonated and Deprotonated Amino Acids and Methylmercury Chalcogenolate Complexes^a^

	R	RCox	TSox	PCox	Pox
Cys	0.00	–6.01	17.84 (23.85)	–47.88	–38.84
Sec	0.00	–6.02	14.06 (20.08)	–40.04	–29.64
Tec	0.00	–6.04	6.25 (12.29)	–48.51	–37.72
Cys^–^	0.00	–19.86	–13.01 (6.85)	–65.14	–50.17
Sec^–^	0.00	–18.7	–13.51 (5.19)	–59.92	–44.08
MeHgCys	0.00	–8.61	12.79 (21.40)	–47.09	–33.81
MeHgSec	0.00	–8.44	9.76 (18.20)	–39.38	–24.33
MeHgTec	0.00	–7.82^b^	3.89 (11.71)	–44.37	–28.68

a For Cys, Sec, Tec, MeHgCys, MeHgSec, and MeHgTec, the reaction evolves to the corresponding chalcogenoxides, while for Cys^–^ and Sec^–^, to deprotonated chalcogenenic acids. Activation energies relative to the RC are given in parentheses. Level of theory: ZORA–BLYP-D3(BJ)/TZ2P.

b RC for MeHgTec converged in a slightly different conformation for H_2_O_2_ with respect to MeHgCys and MeHgSec.

**Figure 2:**
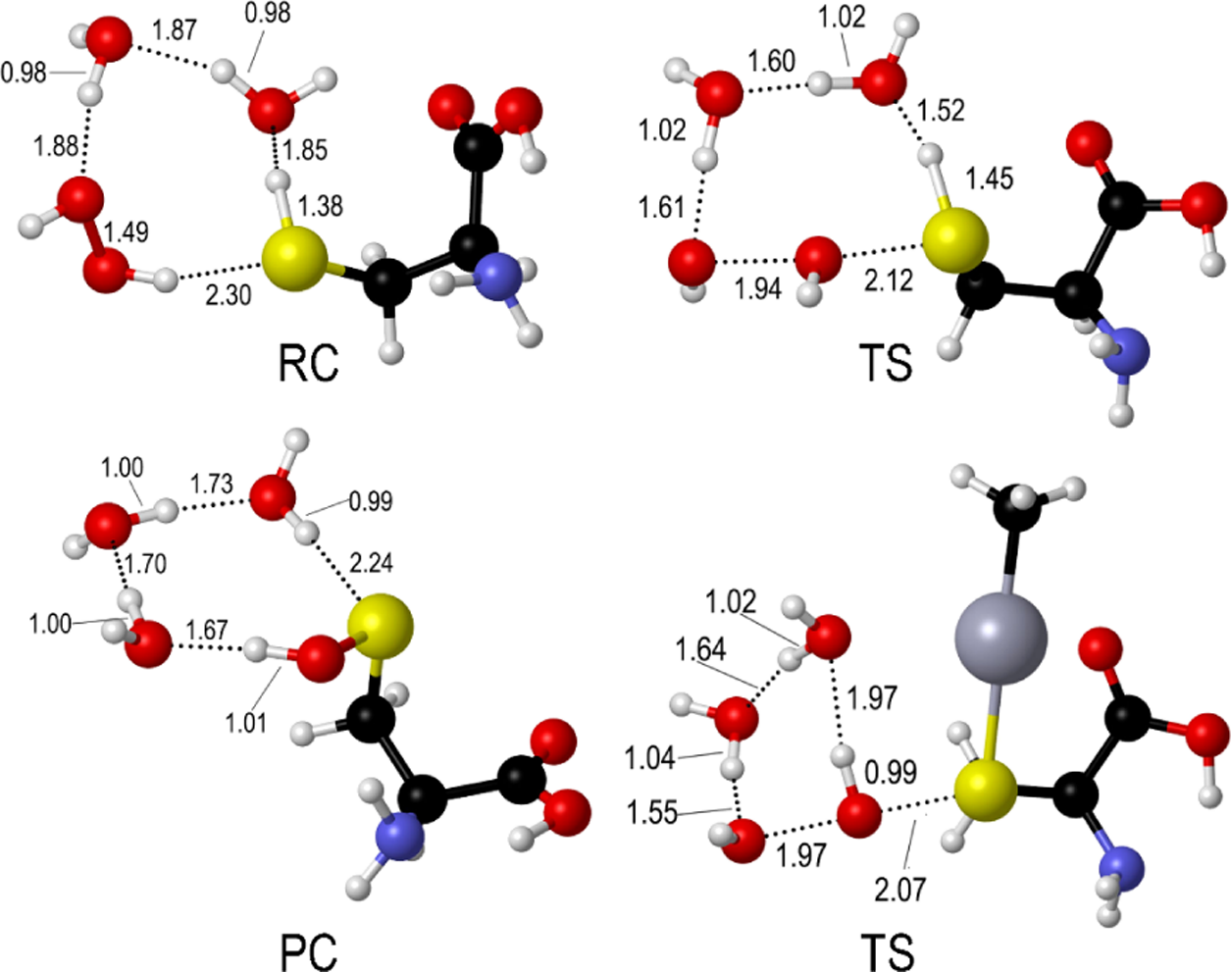
Selected structures for Cys oxidation along the SAPE pathway. (Top left) RC; (top right) TS; (bottom left) PC; (bottom right) TS for MeHgCys oxidation. Relevant interatomic distances are given in Å. Analogous structures were optimized for Sec and MeHgSec.

For Cys and Sec only, the transition states along the SAPE mechanism have been located. All of the attempts to find the analogous TS for Tec failed, suggesting similarity of Tec behavior inside the enzyme and in the free form.^28^ Similar structures were located for Cys and Sec, with a TS state connecting an RC to a PC. At the TS, the X–H (X = S, Se) bond is elongated, indicating partial deprotonation and proton shuttling through a hydrogen-bond network involving Cys, H_2_O_2_, and two water molecules, while the peroxide O–O bond is breaking apart. For methylmercury complexes, similar structures were located. In this case, however, the reaction evolves toward the chalcogenoxides and the complex does not participate in the hydrogen-bond network, which involves only the two water molecules and H_2_O_2_ (Figure 2).

The SAPE mechanism reduces the activation energy required for the oxidation of both free amino acids and the methylmercury complexes (Table 2 vs Table 1), leading to more feasible barriers, as it was previously described for Cys, for which SAPE proved to be a valuable mechanism to reproducing experimentally detected activation energies.^25^

**Table 2:** Electronic Energies (kcal mol^–1^) Relative to Free Reactants for the Oxidation of Free Amino Acids and Methylmercury Chalcogenolate Complexes along the SAPE Pathway^a^

	R	RC	TS	PC	P
Cys	0.00	–22.92	–12.65 (10.27)	–80.81	–49.83
Sec	0.00	–22.43	–15.67 (6.77)	–84.88	–55.42
MeHgCys	0.00	–23.86	–16.54 (7.33)	–67.25	–33.81
MeHgSec	0.00	–23.29	–19.73 (3.65)	–61.09	–24.33

a For Cys and Sec, the reaction evolves to the corresponding chalcogenenic acids, while for MeHgCys and MeHgSec, to the chalcogenoxides. Activation energies relative to the RC are given in parentheses. Level of theory: ZORA–BLYP-D3(BJ)/TZ2P.

For what concerns the anionic mechanism for Cys and Sec, oxidation proceeds in a single step from chalcogenolates to deprotonated chalcogenenic acids, with a transition state connecting a reactant complex to a product complex that closely resembles those of the minimal pathway. In this case, in the RC, hydrogen peroxide assumes a trans-like conformation as shown in Figure 1; however, it is significantly closer to the amine function. Moreover, with respect to the minimal mechanism, in the TS, hydrogen peroxide is far less distorted. Such mechanism displays the lowest activation energy of all of the three under investigation for Cys and Sec oxidation, in agreement with the enhanced nucleophilicity of deprotonated chalcogenols.

### 3.2 Methylmercury Effect

Analogous pathways have been investigated for both free and complexed amino acids, and so information about the influence of CH_3_Hg^+^ binding on the chalcogen nucleus oxidation can be obtained. The results are reported in Table 1 for the minimal and anionic mechanism, and in Table 2 for the SAPE mechanism.

**Figure 3:**
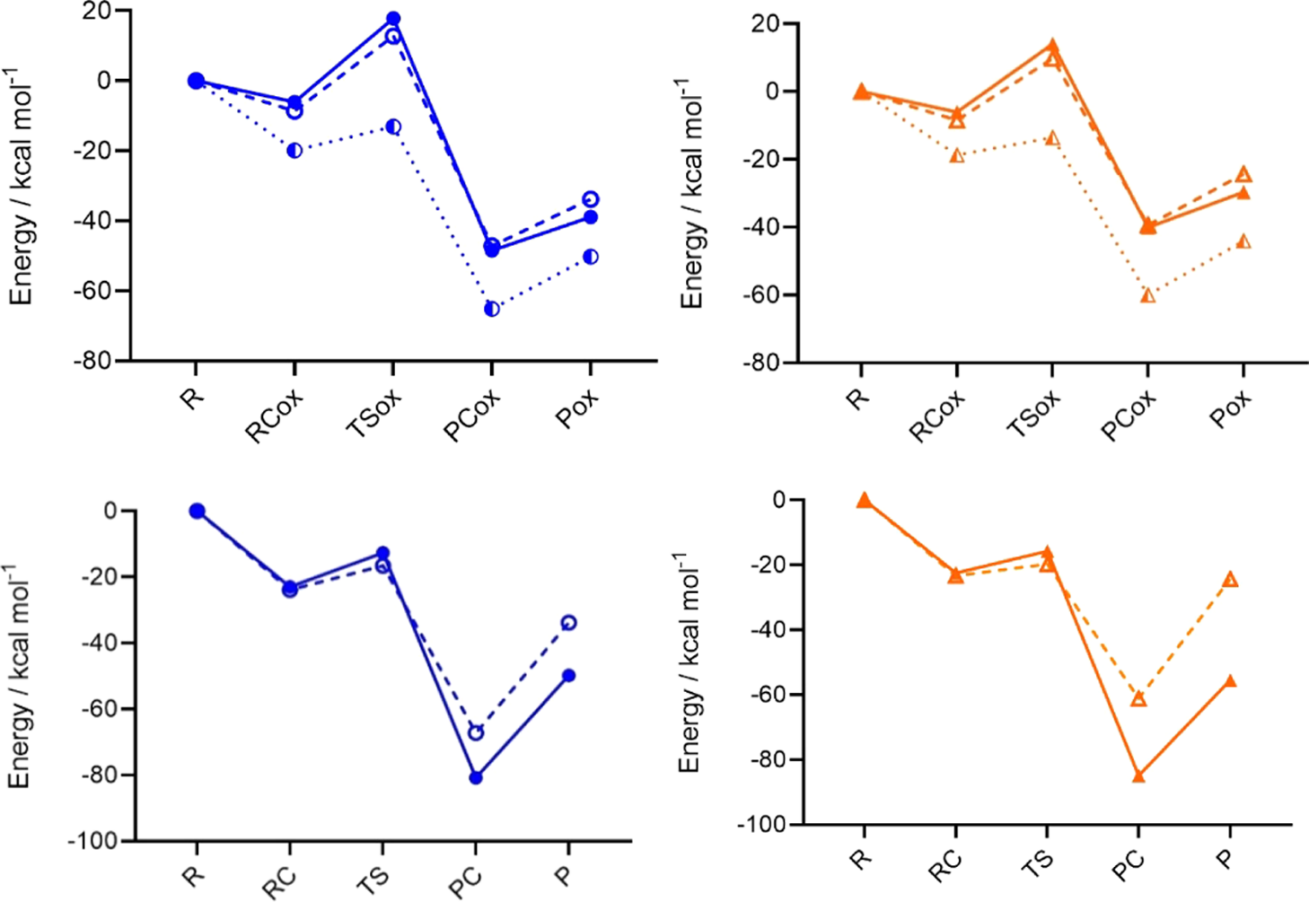
Stationary points for Cys (blue, solid, filled circles), MeHgCys (blue, dashed, void circles), Cys^–^ (blue, dotted, half-filled circles), Sec (orange, solid, filled triangles), MeHgSec (orange, dashed, void triangles), and Sec^–^ (orange, dotted, half-filled triangles); oxidation along the minimal/anionic (top) and SAPE (bottom) pathways. Level of theory: ZORA–BLYP-D3(BJ)/TZ2P.

The replacement of −H with −HgCH_3_ leads to a systematic decrease of the activation energy required for the oxidation. For Cys and Sec, which are the biologically relevant systems, CH_3_Hg^+^ binding leads to a moderate but appreciable decrease in activation energy of about 2–3 kcal mol^–1^ in both mechanisms, which is comparable to the rather modest substituent effect for dichalcogenides oxidations studied by some of us.^41^ Methylmercury chalcogenolates display slightly less negative reaction energies (i.e., less favored reactions). This is likely because the complexes retain their conformation after oxidation, while the free amino acids slightly rearrange. This effect is more prominent along the SAPE pathway, because in this case, the products themselves already show a different stability, with the acids being at a more negative energy with respect to the corresponding oxides. Thus, this is the determinant factor decreasing energetic feasibility (Figure S1). However, all reactions display a prominent negative reaction energy (Figure 3).

The comparison of the anionic pathway (Cys^–^ and Sec^–^) and the minimal pathway (MeHgCys and MeHgSec) shows an opposite trend (Table 1 and Figure 3). With respect to fully deprotonated amino acids, CH_3_Hg^+^ binding significantly increases the activation energy required for peroxide reduction, as it is expected because the chalcogenolates lose the negative charge that promotes an S_N_2-like reaction. Thus, methylmercury chalcogenolate complexes are predicted to reduce hydrogen peroxide at a lower rate than the fully deprotonated amino acids, but anyway faster than the respective protonated amino acids.

### 3.3 Activation Strain Analysis and Methylmercury Effect

The activation strain model of chemical reactivity (or activation strain analysis, ASA) has been employed as described in the computational methods to gain insight into transition state stabilization of the methylmercury complexes with respect to the protonated amino acids. The same method has been successfully applied in precedent studies on dichalcogenides^41^ and chalcogenols.^24^ First, the minimal mechanism will be discussed, since similar conclusions can be drawn for all of the systems.

**Figure 4:**
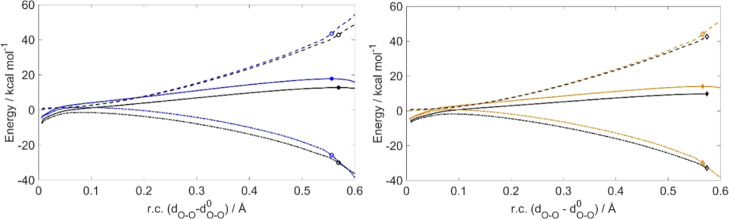
Left: ASA along the rc for the oxidation of Cys (blue) and MeHgCys (black). Right: ASA along the rc for the oxidation of Sec (orange) and MeHgSec (black). The solid lines represent IRC profiles, the dashed lines represent Δ*E*_strain,_ while the dashed-dotted lines represent Δ*E*_int._ Filled symbols (circles, S; diamonds, Se) represent the position of the TS along the rc. Empty symbols represent the value of strain/interaction at the TS. d_O–O_^0^ refers to the O–O bond length in the RC of each reaction.

ASA along the whole reaction coordinate, using IRC geometries, has been carried out for the minimal mechanism of (MeHg)Cys, (MeHg)Sec (Figure 4), and (MeHg)Tec (Figure S2). The system has been partitioned into two fragments, i.e., the chalcogenol/complex and the peroxide, to relate the trends in reactivity to the reactant properties. Then, for both the minimal and the SAPE models, we performed ASA and EDA at the RC and TS.

It is straightforward to assess that methylmercury chalcogenolates undergo faster oxidation mainly because of a larger stabilizing interaction energy. Even if for MeHgCys and MeHgSec the transition state occurs slightly later along the rc, this does not affect significantly the reactivity of these systems, since the strain profiles are almost completely superimposed to those of Cys and Sec. The rather limited influence of replacing −H with −HgCH_3_ on Δ*E*_strain_ was expected, since for this kind of reaction, most of the strain is due to H_2_O_2_ deformation, which undergoes the same structural modifications when changing the substrates. Thus, further analyses have been done as single-point ASA and EDA at the TS and RC.

From EDA (Tables S7 and S8), it emerges that the larger stabilizing Δ*E*_int_ for the complexes is due to the interplay between Δ*E*_Pauli,_ Δ*E*_oi_, and Δ*V*_elstat._ Changing the substituent from −H to −HgCH_3_ leads to a decrease in Pauli repulsion (which becomes less destabilizing) and to a decrease (in absolute value) in orbital interaction and electrostatic interaction (that becomes less stabilizing). In the end, the less stabilizing Δ*E*_oi_ is overcome by the less destabilizing Δ*E*_Pauli_, which leads to a more stabilizing Δ*E*_int_ and thus to a lower-energy TS for MeHgX with respect to X.

As it has been extensively investigated for chalcogenols and dichalcogenides, the activation energy of these systems with hydrogen peroxide correlates with the energy of Cys/Sec HOMO.^24,41^ MeHg(Cys/Sec) displays a higher-energy HOMO, which leads to a less favored energy match to H_2_O_2_ LUMO responsible for the less stabilizing Δ*E*_oi_, since at the transition state, the LUMO of H_2_O_2_ has a lower energy with respect to the HOMO of the substrate^62^ (Figure S3). However, even if Δ*E*_oi_ becomes less stabilizing, as a rule of thumb, the higher the HOMO, the faster the reactivity with H_2_O_2_. Similar conclusions can be drawn when comparing MeHgTec to Tec oxidation (Table S9) and is thus a general effect of CH_3_Hg^+^ binding, which destabilizes Cys/Sec/Tec HOMOs and lowers Δ*E*_Pauli._

**Figure 5:**
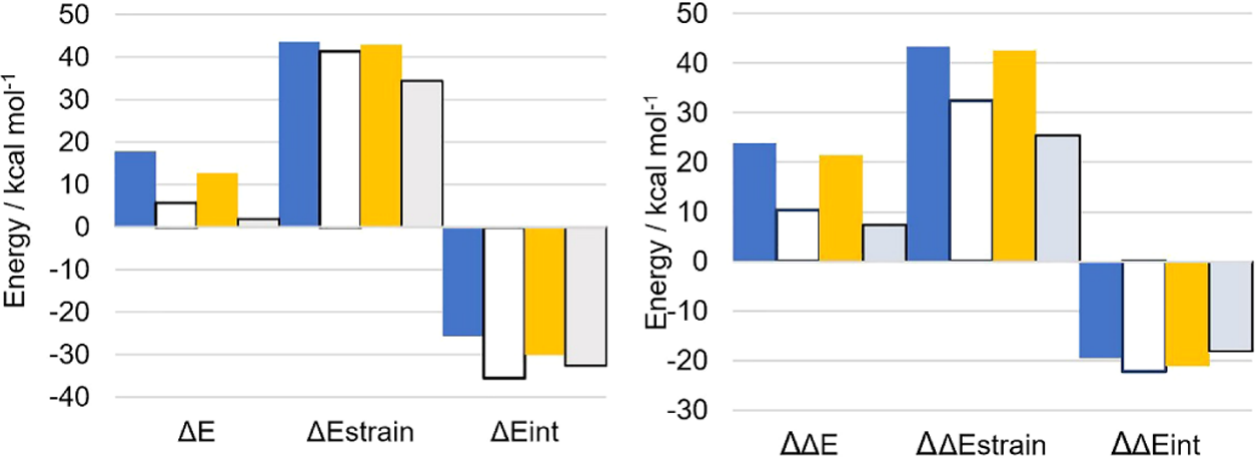
ASA for Cys (blue), Cys, SAPE mechanism (white), MeHgCys (orange), and MeHgCys, SAPE mechanism (gray). Right: relative variations of energy values (TS–RC); left: the energy values at the transition state.

**Figure 6:**
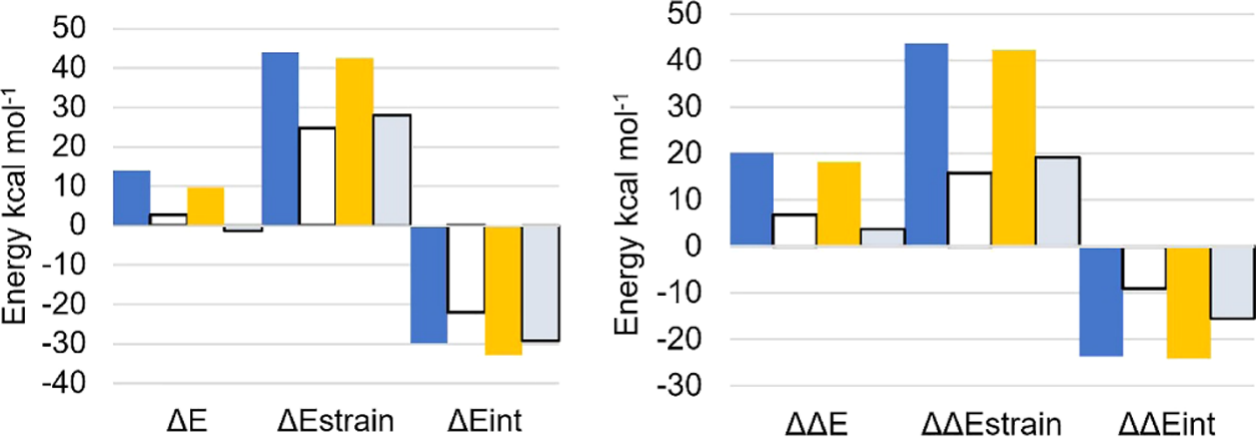
ASA for Sec (blue), Sec SAPE mechanism (white), MeHgSec (orange), and MeHgSec, SAPE mechanism (gray). Right: relative variations of energy values (TS–RC); left: the energy values at the transition state.

Along the SAPE mechanism, discrepancies arise both from the exchange of −H with −HgCH_3_ and from mechanistic differences that lead to different products (chalcogenenic acids and chalcogenoxides, respectively). Thus, from our ASA, a different picture can be seen with respect to the minimal model, both for Cys (Figure 5) and for Sec (Figure 6). Moreover, while the definition of the fragments for the minimal model is straightforward, for the SAPE mechanism, it is less trivial. Particularly, to avoid uncommon negative strain energies, we partitioned the system into the chalcogenol/complex and a fragment composed of two water molecules and hydrogen peroxide. We set as a reference point for relative energies the chalcogenol/complex and a fictitious reactant formed after optimization of a ring composed of two water molecules and hydrogen peroxide only (Table S1, fictitious reactant). The trends are consistent with those obtained using as a reference point the free reactants (Table S10), and discrepancies in the energies of RCs and TSs with respect to Table 2 arise from the different choice of the reference state.

For the SAPE oxidation, it is immediately possible to note how MeHgCys undergoes faster oxidation with respect to Cys because of an important lowering of Δ*E*_strain_ from RC to TS, despite a less stabilizing Δ*E*_int_. This effect has been attributed to the mechanistic differences between the two reactions, with Cys displaying a higher Δ*E*_strain_ because of S–H bond deformation, which is breaking apart at the transition state. Conversely, MeHgCys does not undergo any major distortion during the reaction, because it is not involved in the SAPE hydrogen-bond network, and this reflects into the lower activation strain required for the process to occur. The difference in Δ*E*_int_ with respect to the minimal mechanism can be rationalized in terms of HOMO–LUMO interaction. MeHgCys still displays a higher HOMO than Cys, as investigated for the minimal mechanism. However, because of the different hydrogen-bond network in Cys and MeHgCys transition states, H_2_O_2_ LUMO for Cys oxidation is significantly higher in energy than in MeHgCys oxidation. In the former case, this reduces the HOMO–LUMO energy gap enough to make Δ*E*_oi_ much more stabilizing for Cys than for MeHgCys. Thus, along the SAPE mechanism, the lowering in Δ*E*_Pauli_ that occurs replacing −H with −HgCH_3_ is not enough to compensate for the loss in Δ*E*_oi_, leading to a less stabilizing interaction energy for MeHgCys.

An opposite scenario can be seen for Sec and MeHgSec SAPE oxidation. In this case, MeHgSec displays a more stable TS because of a more stabilizing interaction energy and despite a more destabilizing strain energy (Figure 6).

In this case, this effect is ascribed to the different position along the rc for the two TSs. In fact, while for Cys and MeHgCys, the two transition states occur in close proximity along the rc (with differences on O–O bond of 0.03 Å, and MeHgCys displaying a slightly later transition state similarly to the minimal mechanism), Sec displays a significantly earlier transition state with respect to MeHgSec (with d_O–O_ of 1.77 and 1.91 Å, respectively). Thus, the higher activation strain required to reach MeHgSec TS follows naturally from the more severe deformation of H_2_O_2_, while the more negative Δ*E*_int_ is at least partly a consequence of the closer distance between the two interacting fragments, which in the end leads to a lower-energy TS. Comparing Figure 6 with Figure 5, it is possible to note that with respect to Cys, Sec displays a far less stabilizing Δ*E*_int_, while MeHgCys and MeHgSec show close values of Δ*E*_int_, thus enhancing our confidence in linking the trend differences between the two amino acids and complexes to the relative position along the rc at which their TSs occur.

Finally, ASA (Table 3) and EDA (Table S11) have been performed on the anionic system (Cys^–^ and Sec^–^) also, and the results have been compared to the minimal oxidation of MeHgCys and MeHgSec. Along the anionic pathway, the transition state is reached far earlier along the rc, with d_O–O_ around 1.70 Å, while along the minimal pathway, d_O–O_ is around 2.00 Å in both cases.

**Table 3:** ASA (kcal mol^–1^) for Cys^–^ and Sec^–^ and for MeHgCys and MeHgSec^a^

		Δ*E*	Δ*E*_strain_	Δ*E*_int_
Cys^–^	RC	–19.86	3.25	–23.11
	TS	–13.01	9.52	–22.53
Sec^–^	RC	–18.70	2.74	–21.44
	TS	–13.51	8.45	–21.96
MeHgCys	RC	–8.61	0.29	–8.90
	TS	12.79	42.84	–30.05
MeHgSec	RC	–8.44	0.31	–8.75
	TS	9.76	42.63	–32.87

a Level of theory: ZORA–BLYP-D3(BJ)/TZ2P.

This affects mainly the strain energy, which is much lower when going from RC to TS along the anionic pathway with respect to the minimal pathway, where stronger interaction energies can be seen. This effect is well documented in the literature and is rooted in the shape of the interaction energy curve.^52^ In fact, stronger nucleophiles (i.e., negatively charged in our case) display stronger interaction energies along the whole rc, even if at the TS alone, the analysis can misleadingly suggest otherwise, as it is in our system, because of the different point along the rc at which the analysis has been performed. In fact, for the minimal mechanism (Figure 3, left) at rc = 0.21 Å corresponding to the TS of Cys^–^, Δ*E*_int_ of MeHgCys is computed to be around −4.00 kcal mol^–1^, far less stabilizing than that of Cys^–^. Thus, we conclude that methylmercury chalcogenolates display later TSs with a higher energy with respect to free chalcogenolates because of a shallower interaction energy curve, in line with the Hammond postulate.^51^

## 4 Conclusions

In this work, we have investigated *in silico* how the peroxide-reducing potential of Cys, Sec, and Tec is affected by CH_3_Hg^+^ binding. Almost certainly, inside a catalytic pocket designed to promote a peroxidatic behavior of Cys and Sec, CH_3_Hg^+^ lowers the rate of the oxidative step by inhibiting the proton transfer mechanism and thus blocking the formation of a charge-separated intermediate. While this pathway has not been directly investigated, we have found that with respect to negatively charged chalcogenolates, methylmercury complexes display an importantly lower reactivity, as it is expected because of the weakening of the nucleophilic power of the chalcogen nucleus. A canonical Hammond behavior has been observed that fully explains such a change in reactivity. Since the catalytically active Sec of GPx reacts even faster than a fully deprotonated Sec, the same trend is expected in the enzyme.

Moreover, our models predict methylmercury (seleno)cysteinate to be more readily oxidized than the protonated free amino acids. The reasons accounting for this reactivity have been explained by different mechanisms and are different when changing the chalcogen to which CH_3_Hg^+^ binds. However, a few general insights are obtained. First, methylmercury chalcogenolates display a destabilized HOMO with respect to the free protonated amino acids. Thus, in agreement with previous studies on the oxidation of organochalcogen compounds, the higher the HOMO of the substrate, the higher its reactivity toward H_2_O_2_. Second, while for SAPE oxidation such correlation still holds true, along this pathway, trends in activation energies are related to the intrinsically different participation of the substrate in the hydrogen-bond network, which in turn affects either directly the strain energy or indirectly the position along the rc, where the TS occurs.

Based on the knowledge that arylated (i.e., inhibited) Sec can be readily oxidized by H_2_O_2_,^63^ and knowing that CH_3_Hg^+^ displays a substituent effect similar to that of alkyl/aryl substituents on diselenides,^41^ our calculations suggest that, after CH_3_Hg^+^ binding, the catalytically relevant chalcogen nucleus might still reduce one equivalent of peroxide, even if at a significantly lower rate with respect to the active enzyme and leading to a chalcogenoxide instead of a chalcogenenic acid moiety. Thus, after CH_3_Hg^+^ binding, the oxidation to chalcogenoxide might make new reactions feasible, such as selenoxide elimination, which has been hypothesized to be responsible for the irreversible inactivation of GPxs under highly oxidizing conditions,^64^ and it is involved in the irreversible inactivation of small-molecule-inhibited TrxR.^63^ Alternatively, further evolution of the selenoxide might lead to the insertion of the oxygen atom between the chalcogen and mercury atom, leading to a structure similar to the one hypothesized for PhSeZnCl oxidation. Also, in that case, the presence of zinc increased the catalytic activity of diphenyl diselenide toward thiol oxidation by hydrogen peroxide.^65^ Our study prompts a more detailed investigation of methylmercury chalcogenolates reactivity. Experimental investigation of our trends, both at the single residue and at the enzymatic level, might help reach a better understanding of the evolution of CH_3_Hg^+^-toxified enzymes and of the chemical details behind selenoproteins inhibition by methylmercury.

## Supporting Information Available

Cartesian coordinates, electronic energies, and imaginary frequencies of the investigated compounds; Gibbs free energies and energies in water; and additional ASA and EDA calculations (PDF)
