# Supplementary material for: Effect of Methylmercury Binding on the Peroxide-Reducing Potential of Cysteine and Selenocysteine
Source: Inorg Chem. 2021 Feb 15;60(7):4646–56. doi: 10.1021/acs.inorgchem.0c03619 (PMC8763373; doi:10.1021/acs.inorgchem.0c03619)

♦ SUPPORTING INFORMATION ♦

Effect of Methylmercury Binding on the Peroxide Reducing Potential of Cysteine and Selenocysteine

Andrea Madabeni,[a] Pablo A. Nogara,[a,b] Marco Bortoli,[a] Joao B. T. Rocha,[b] Laura Orian[a*]

[a] Dipartimento di Scienze Chimiche, Università degli Studi di Padova, Via Marzolo 1, 35131, Padova, Italy

[b] Departamento de Bioquímica e Biologia Molecular, Universidade Federal de Santa Maria, Santa Maria RS Brazil

[*] Corresponding author: E-mail: laura.orian@unipd.it

Table of Contents

Table S1. Coordinates (Å) energies (E, Hartree) and number of imaginary frequencies (Nimag) of stationary points as computed at ZORA-BLYP-D3(BJ)/TZ2P level of theory. S4

Table S2. Activation energies (kcal mol^{-1}) relative to RC for Cys, Sec, MeHgCys and MeHgSec (SAPE mechanism) in gas phase. Level of theory ZORA-B3LYP-D3(BJ)/TZ2P // ZORA-B3LYP/TZ2P. S16

Table S3. Energies (kcal mol^{-1}) relative to free reactants for the minimal oxidation mechanism of (MeHg)X, where (X=Cys, Sec), and anionic mechanism (Cys^-, Sec^-) in water. Level of theory COSMO-ZORA-BLYP-D3(BJ)/TZ2P // ZORA-BLYP-D3(BJ)/TZ2P. S16

Table S4. Energies (kcal mol^{-1}) relative to free reactants for the SAPE RC and TS of Cys, Sec, MeHgCys and MeHgSec in water. Level of theory COSMO-ZORA-BLYP-D3(BJ)/TZ2P // ZORA-BLYP-D3(BJ)/TZ2P...........S16

Table S5. Gibbs free energies (kcal mol^{-1}) relative to free reactants for the minimal and anionic mechanisms, gas phase. Level of theory ZORA-BLYP-D3(BJ)/TZ2P. S16

Table S6. Gibbs free energies (kcal mol^{-1}) relative to free reactants for the SAPE oxidation mechanism of (MeHg)X, where (X=Cys, Sec) in gas phase. Level of theory ZORA-BLYP-D3(BJ)/TZ2P.............................S17

Table S7. ASA-EDA for the stationary points for Cys and MeHgCys oxidation on both mechanisms studied (minimal and SAPE). Energies (kcal mol^{-1}) relative to Cys/MeHgCys and H_2O_2 (minimal) and fictitious reactant (SAPE mechanism). Level of theory ZORA-BLYP-D3(BJ)/TZ2P S17

Table S8. ASA-EDA for the stationary points for Sec and MeHgSec oxidation on both mechanisms studied (minimal and SAPE). Energies (kcal mol^{-1}) relative to Cys/MeHgCys and H_2O_2 (minimal) and fictitious reactant (SAPE mechanism)**.** Level of theory ZORA-BLYP-D3(BJ)/TZ2P S17

Table S9. ASA-EDA for the stationary points for Tec and MeHgTec minimal oxidation. Energies (kcal mol^{-1}) relative to Tec/MeHgTec and H_2O_2 fragments. Level of theory ZORA-BLYP-D3(BJ)/TZ2P S18

Table S10. ASA comparison for Cys, Sec, MeHgCys, MeHgSec (SAPE mechanism) employing as reference state the free reactants or the fictitious reactant. Energies in kcal mol^{-1}. Level of theory ZORA-BLYP-D3(BJ)/TZ2P. S18

Table S11. EDA for Cys^- and Sec^- (anionic mechanism) employing as reference state the free reactants. Energies in kcal mol^{-1}. Level of theory: ZORA-BLYP-D3(BJ)/TZ2P. S18

Figure S1. Stationary points for the stepwise oxidation of Cys (blue, solid), Sec (orange, dashed) and Tec (black, dashed-dotted). Level of theory ZORA-BLYP-D3(BJ)/TZ2P. S19

Figure S2. ASA along the r.c. for the oxidation of Tec (blue) and MeHgTec (black). Solid lines represent IRC profiles, dashed lines represent $\Delta E_{strain,}$ while dashed-dotted lines represent $\Delta E_{int.}$ $d^0_{O\text{-}O}$ refers to the O-O bond length in the RC. Level of theory ZORA-BLYP-D3(BJ)/TZ2P S19

Figure S3. Molecular orbitals of (MeHg)Cys and of H_2O_2 in the geometry they possess at the transition states of the respective oxidation reactions, mainly responsible for ΔE_{oi}. Isosurface value 0.05 a.u. Level of theory: ZORA-BLYP-D3(BJ)/TZ2P. *Top* Cys transition state geometry. Left: Cys HOMO; right: H_2O_2 LUMO. *Bottom*: MeHgCys transition state geometry. Left: MeHgCys HOMO; right: H_2O_2 LUMO............S20

Table S1. Coordinates (Å) energies (E, Hartree) and number of imaginary frequencies (Nimag) of stationary points as computed at ZORA-BLYP-D3(BJ)/TZ2P level of theory.

R

H_2O_2
Nimag=0
E=-0.64253188

O	-0.950665	-3.866755	-0.829678
H	-1.218280	-2.948615	-0.622989
H	-1.584416	-4.334246	-2.577082
O	-0.836332	-3.759752	-2.317018

Cys
Nimag=0
E= -2.77711318

C	1.336988	-3.312415	7.397812
C	2.655104	-3.484665	6.620510
N	1.473042	-2.906189	8.811379
H	2.319804	-3.326719	9.206055
H	1.541955	-1.894695	8.909985
H	0.742869	-2.543712	6.882541
C	0.476442	-4.607303	7.312179
O	0.410983	-5.296513	6.315779
O	-0.212664	-4.870886	8.445941
H	0.084463	-4.162155	9.082877
H	2.450391	-3.831560	5.606030
H	3.188662	-2.530493	6.570423
S	3.839338	-4.651186	7.431308
H	3.129162	-5.773162	7.165605

MeHgCys
Nimag=0
E=-3.35208986

C	-0.622081	0.749421	-0.118287
C	0.752208	0.650549	-0.806758
N	-0.585216	1.100281	1.318356
H	0.278367	0.722141	1.723907
H	-0.590375	2.109021	1.459824
H	-1.209725	1.517155	-0.642887
C	-1.434639	-0.563260	-0.291737
O	-1.443121	-1.218487	-1.318442
O	-2.145265	-0.903121	0.803068
H	-1.887293	-0.204545	1.475327
H	0.623039	0.305988	-1.834215
H	1.220762	1.639768	-0.826847
S	1.955362	-0.440109	0.097593
Hg	1.177645	-2.646800	-0.535481
C	0.547996	-4.608510	-1.077240
H	1.164533	-4.968060	-1.904203
H	0.651285	-5.267948	-0.212329
H	-0.498626	-4.536249	-1.380848

Cys^-
Nimag=0
E= -2.68869302

C	1.276548	-3.125802	7.449938
C	2.636045	-3.280111	6.695914
N	1.440701	-3.028616	8.926588
H	2.385395	-3.455277	9.095213
H	1.479484	-2.050474	9.215854
H	0.768054	-2.221044	7.081602
C	0.291237	-4.280909	7.142480
O	-0.040905	-4.619923	6.019228
O	-0.236779	-4.839495	8.263100
H	0.231455	-4.308388	8.988373
H	2.417469	-3.702544	5.709122
H	3.046194	-2.268490	6.543591
S	3.847173	-4.325186	7.619898

Sec
Nimag=0
E=-2.74303072

C	1.327457	-3.297536	7.396030
C	2.635073	-3.446550	6.605685
N	1.464252	-2.895201	8.810822
H	2.316054	-3.309624	9.202286
H	1.522945	-1.883440	8.913280
H	0.720575	-2.531456	6.890150
C	0.473943	-4.597346	7.305826
O	0.399055	-5.274062	6.301930
O	-0.200965	-4.876742	8.444439
H	0.095887	-4.170546	9.084276
H	2.438110	-3.815448	5.599105
H	3.168294	-2.493563	6.552617
Se	3.946623	-4.698024	7.466538
H	3.129237	-5.902114	7.185438

MeHgSec
Nimag=0
E=-3.32769286

C	-0.628858	0.756844	-0.120493
C	0.738256	0.713923	-0.817078
N	-0.609396	1.121001	1.313308
H	0.260271	0.768902	1.730051
H	-0.642423	2.130466	1.445939
H	-1.251309	1.497217	-0.646232
C	-1.393482	-0.585615	-0.285336
O	-1.376278	-1.248942	-1.306595
O	-2.098158	-0.939359	0.808796
H	-1.868386	-0.227019	1.476446
H	0.634022	0.341098	-1.835711
H	1.183174	1.712191	-0.840104
Se	2.099128	-0.415932	0.154832
Hg	1.216952	-2.701482	-0.524567
C	0.512814	-4.640772	-1.087833
H	1.119394	-5.014686	-1.915645
H	0.587777	-5.309284	-0.227138
H	-0.528643	-4.521318	-1.393839

Sec^-
Nimag=0
E= -2.66591724

C	1.320280	-3.298301	7.377780
C	2.669233	-3.484818	6.640395
N	1.460488	-2.966056	8.823500
H	2.404357	-3.333887	9.086313
H	1.473477	-1.953743	8.954173
H	0.757122	-2.485979	6.889089
C	0.391410	-4.534089	7.246674

O	0.103540	-5.066596	6.189106
O	-0.151336	-4.916286	8.432170
H	0.279611	-4.261073	9.071891
H	2.489130	-4.047194	5.721301
H	3.067172	-2.496731	6.377016
Se	4.042820	-4.444785	7.763575

Tec
Nimag=0
E= -2.71115909

C	1.312767	-3.276318	7.397401
C	2.613290	-3.399513	6.592721
N	1.446653	-2.875250	8.813049
H	2.298441	-3.286834	9.209274
H	1.502940	-1.863345	8.916044
H	0.690031	-2.515578	6.901594
C	0.471860	-4.583981	7.303993
O	0.396687	-5.254283	6.296200
O	-0.195662	-4.876316	8.444337
H	0.093668	-4.169306	9.086181
H	2.415320	-3.779783	5.590733
H	3.129717	-2.438136	6.528814
Te	4.099948	-4.760932	7.499217
H	3.160879	-6.112077	7.178863

MeHgTec
Nimag=0
E=-3.30304773

C	-0.638778	0.775856	-0.122956
C	0.729656	0.785549	-0.813976
N	-0.658140	1.180929	1.299902
H	0.208569	0.864147	1.750549
H	-0.718845	2.192742	1.401876
H	-1.295854	1.471067	-0.669420
C	-1.339249	-0.604013	-0.259487
O	-1.273334	-1.296894	-1.258938
O	-2.052988	-0.954203	0.829968
H	-1.870552	-0.213874	1.480533
H	0.652769	0.386932	-1.824941
H	1.136209	1.799080	-0.850579
Te	2.282155	-0.398954	0.251801
Hg	1.266921	-2.806418	-0.511645
C	0.480203	-4.712611	-1.120387
H	1.032264	-5.059966	-1.996511
H	0.586285	-5.419791	-0.294694
H	-0.572435	-4.552344	-1.362296

Pox

H_2O
Nimag=0
E=-0.5067482

O	-0.812277	-3.797985	0.
H	-0.555031	-2.860026	0.
H	-1.784755	-3.783047	0.

Cys
Nimag=0
E=-2.97478914

C	1.219518	-3.412358	7.235030
C	2.317833	-3.972441	6.314628
N	1.689169	-2.587861	8.371365
H	2.640681	-2.850216	8.644687
H	1.675102	-1.594468	8.151095
H	0.558696	-2.795973	6.611353
C	0.299529	-4.561234	7.757496
O	-0.077734	-5.473075	7.050839
O	-0.051069	-4.412959	9.050832
H	0.448509	-3.594016	9.341261
H	1.879268	-4.593273	5.526053
H	2.921739	-3.166633	5.887720
S	3.467057	-5.094622	7.267420
O	4.204541	-4.233816	8.275005
H	4.280926	-5.223871	6.140630

MeHgCys
Nimag=0
E=-3.54175909

C	-0.709410	0.761523	-0.191041
C	0.660081	0.428235	-0.797029
N	-0.679395	1.523430	1.079078
H	0.122125	1.231321	1.644130
H	-0.596911	2.524962	0.912180
H	-1.269054	1.363641	-0.921252
C	-1.585283	-0.508580	0.009257
O	-1.568997	-1.461823	-0.748705
O	-2.393686	-0.440060	1.085433
H	-2.131706	0.416525	1.531235
H	0.561669	-0.036567	-1.779209
H	1.275614	1.329503	-0.881368
S	1.674760	-0.655887	0.386303
Hg	1.053918	-2.875840	-0.668009
C	0.610394	-4.795599	-1.548762
H	1.145409	-4.870601	-2.498033
H	0.936254	-5.576850	-0.858625
H	-0.469799	-4.834570	-1.703967
O	3.144687	-0.347895	0.109799

Cys-
Nimag=0
E= -2.66591724

C	1.362862	-3.030206	7.264585
C	2.591978	-3.287748	6.373898
N	1.700728	-2.371771	8.561651
H	2.755139	-2.406371	8.686118
H	1.419078	-1.392669	8.547120
H	0.634809	-2.408002	6.709706
C	0.590896	-4.344410	7.583214
O	0.189723	-5.126828	6.735256
O	0.344175	-4.481731	8.903355
H	0.829550	-3.649167	9.266113
H	2.341897	-4.022150	5.598506
H	2.903054	-2.341839	5.900130
S	4.029149	-3.947684	7.341451
O	4.403337	-2.701920	8.315072

Sec
Nimag=0
E=-2.92604446

C	1.193972	-3.409378	7.227149
C	2.247004	-3.947206	6.249011
N	1.702005	-2.585273	8.344332

H	2.626952	-2.923646	8.639428
H	1.774438	-1.602923	8.087326
H	0.490996	-2.796993	6.643746
C	0.314507	-4.565335	7.796958
O	-0.004562	-5.537453	7.141788
O	-0.069133	-4.356240	9.071290
H	0.417923	-3.518775	9.331533
H	1.791780	-4.526913	5.441591
H	2.878598	-3.147266	5.859273
Se	3.491908	-5.246914	7.213098
O	4.095078	-4.327883	8.494190
H	4.522297	-5.074619	6.084701

MeHgSec
Nimag=0
E=-3.50224155

C	-0.738298	0.771035	-0.184105
C	0.635068	0.449565	-0.772397
N	-0.736792	1.598126	1.046955
H	0.080865	1.382246	1.622246
H	-0.706205	2.592430	0.827676
H	-1.319359	1.320277	-0.940498
C	-1.575789	-0.514607	0.078293
O	-1.534290	-1.562	-0.635683
O	-2.377510	-0.423740	1.158438
H	-2.142870	0.461909	1.560577
H	0.559103	-0.074526	-1.724472
H	1.249354	1.346395	-0.885911
Se	1.782174	-0.690567	0.519677
Hg	1.087384	-2.972782	-0.636513
C	0.597331	-4.840691	-1.612399
H	1.166754	-4.899999	-2.542376
H	0.864222	-5.658917	-0.940230
H	-0.476776	-4.825211	-1.807560
O	3.366302	-0.346012	0.029698

Sec^-
Nimag=0
E= -2.87194672

C	1.352201	-3.028930	7.254408
C	2.547877	-3.287091	6.328612
N	1.713519	-2.374388	8.545424
H	2.777564	-2.409692	8.673824
H	1.429466	-1.395910	8.540540
H	0.611271	-2.405440	6.719220
C	0.579877	-4.341635	7.586946
O	0.167661	-5.126415	6.745784
O	0.351562	-4.469632	8.909059
H	0.849598	-3.633149	9.257762
H	2.278979	-3.999321	5.541680
H	2.895959	-2.345172	5.886613
Se	4.120141	-4.044395	7.357037
O	4.420701	-2.651327	8.439267

Tec
E= -2.90689674
Nimag=0

C	1.185138	-3.397467	7.257164
C	2.207921	-3.924926	6.237726
N	1.688031	-2.506010	8.317331
H	2.519915	-2.935588	8.745415
H	1.918479	-1.580983	7.958948
H	0.417492	-2.844135	6.693364
C	0.403686	-4.558864	7.931167
O	0.293705	-5.664103	7.431975
O	-0.166857	-4.210906	9.099286
H	0.228607	-3.318908	9.309354
H	1.722484	-4.447792	5.409803
H	2.848913	-3.124894	5.864424
Te	3.513620	-5.427312	7.236421
O	3.544093	-4.685340	8.941200
H	4.899171	-4.693273	6.498421

MeHgTec
E= -3.48452641
Nimag=0

C	-0.755085	0.779211	-0.187118
C	0.638934	0.481302	-0.736580
N	-0.808400	1.660958	1.005152
H	0.006908	1.508134	1.603560
H	-0.813935	2.644563	0.740232
H	-1.343941	1.275115	-0.974436
C	-1.551712	-0.522295	0.118347
O	-1.464382	-1.536326	-0.549875
O	-2.371838	-0.415142	1.183922
H	-2.177755	0.496244	1.548208
H	0.593692	-0.082054	-1.668425
H	1.221169	1.394509	-0.885155
Te	1.939259	-0.709649	0.677442
Hg	1.130032	-3.083545	-0.597145
C	0.548805	-4.889636	-1.653
H	0.988698	-4.856842	-2.649191
H	0.920180	-5.750952	-1.090686
H	-0.541875	-4.892283	-1.700920
O	3.621915	-0.326444	-0.025913

Piso

Cys
Nimag=0
E=-2.99229697

C	1.239662	-3.420215	7.238430
C	2.312816	-4.046366	6.331556
N	1.749187	-2.629992	8.383775
H	2.700235	-2.908430	8.629468
H	1.753455	-1.632279	8.182841
H	0.619033	-2.761025	6.614840
C	0.260300	-4.521864	7.748482
O	-0.187993	-5.387486	7.025724
O	-0.065503	-4.388218	9.051584
H	0.482860	-3.608852	9.355796
H	1.823894	-4.678442	5.579969
H	2.900915	-3.279083	5.815904
S	3.451481	-5.203797	7.181932
O	4.435514	-4.114341	8.080473
H	5.216092	-3.918532	7.528307

Sec
Nimag=0
E=-2.96713264

C	1.217648	-3.416785	7.224398
C	2.260522	-4.035434	6.288237

N	1.760972	-2.638312	8.362469
H	2.719133	-2.920970	8.579417
H	1.762170	-1.639024	8.169318
H	0.582405	-2.748173	6.624422
C	0.245834	-4.516794	7.752245
O	-0.214666	-5.384402	7.038835
O	-0.056503	-4.380	9.059921
H	0.506117	-3.605821	9.353748
H	1.768719	-4.646678	5.524376
H	2.881236	-3.276507	5.804611
Se	3.483490	-5.325616	7.195185
O	4.504170	-4.088802	8.167925
H	5.270701	-3.875606	7.603975

Tec
Nimag=0
E=-2.95172110

C	1.193195	-3.413723	7.213064
C	2.203210	-4.033065	6.244127
N	1.771992	-2.648591	8.343696
H	2.730903	-2.943972	8.544158
H	1.786576	-1.649045	8.151947
H	0.548021	-2.730732	6.639464
C	0.221658	-4.504572	7.758383
O	-0.252523	-5.374768	7.056450
O	-0.064067	-4.358436	9.068802
H	0.513353	-3.591076	9.352627
H	1.689499	-4.615743	5.473979
H	2.834458	-3.277092	5.770674
Te	3.555364	-5.479835	7.199056
O	4.599116	-4.068535	8.240969
H	5.361193	-3.809739	7.691686

RCox

Cys
Nimag=0
E= -3.42922462

C	1.348656	-3.315365	7.319501
C	2.744928	-3.611122	6.750744
N	1.312640	-2.605195	8.614551
H	2.079715	-2.930057	9.209435
H	1.409851	-1.598179	8.494480
H	0.820047	-2.690744	6.584471
C	0.501800	-4.617184	7.422164
O	0.583032	-5.527970	6.624794
O	-0.353475	-4.621624	8.470520
H	-0.147011	-3.778760	8.961728
H	2.667470	-4.136547	5.797771
H	3.308847	-2.686985	6.601353
S	3.795034	-4.621004	7.895782
H	3.160969	-5.796	7.668733
O	6.049198	-3.915546	5.642800
O	5.887970	-2.512687	6.133795
H	6.788840	-2.356782	6.480184
H	5.500218	-4.383308	6.322325

MeHgCys
Nimag=0
E= -4.00834039

C	1.471257	-3.157015	7.383195
C	2.951794	-3.279153	6.980002
N	1.218857	-2.974988	8.828871
H	1.961043	-3.444501	9.358231
H	1.213349	-1.990750	9.090779
H	1.048721	-2.292710	6.850562
C	0.651246	-4.376401	6.876822
O	0.826049	-4.896337	5.789654
O	-0.283594	-4.796417	7.752858
H	-0.142391	-4.204268	8.549843
H	3.023093	-3.510255	5.915820
H	3.463000	-2.329927	7.168113
S	3.887054	-4.541359	7.976021
Hg	3.088647	-6.644223	7.010565
C	2.419789	-8.499314	6.211633
H	1.509008	-8.295109	5.644834
H	3.202872	-8.895738	5.561976
H	2.214329	-9.191091	7.031148
H	5.507509	-4.726403	6.419396
O	5.425388	-6.107873	5.075111
H	5.059433	-5.739169	4.246591
O	6.088035	-4.885847	5.623389

Cys^-
Nimag=0
E= -3.36287681

C	1.419858	-3.047485	7.538881
C	2.719870	-3.244321	6.693697
N	1.634330	-3.057937	9.007515
H	2.462330	-3.677450	9.132603
H	1.958655	-2.137392	9.311623
H	0.969999	-2.082896	7.258622
C	0.323591	-4.091776	7.204590
O	-0.016959	-4.398970	6.075593
O	-0.281988	-4.586344	8.315519
H	0.233234	-4.113736	9.051423
H	2.429072	-3.652502	5.720826
H	3.164186	-2.253374	6.516768
S	3.965763	-4.338685	7.508704
O	3.997915	-0.925719	9.108464
O	5.156870	-1.882295	8.960776
H	4.717717	-2.723194	8.535000
H	3.993817	-0.541056	8.210721

Sec
Nimag=0
E= -3.39516085

C	1.460252	-3.393096	7.123020
C	2.760071	-3.917551	6.505249
N	1.597080	-2.599039	8.361777
H	2.345139	-2.988612	8.942475
H	1.824485	-1.626195	8.162296
H	0.980429	-2.747363	6.371696
C	0.441595	-4.546872	7.357795
O	0.334469	-5.500495	6.616091
O	-0.334514	-4.364533	8.451305
H	0.020578	-3.532311	8.870531

H	2.559626	-4.516315	5.617304
H	3.443922	-3.104322	6.254384
Se	3.821798	-5.068263	7.765754
H	5.388160	-5.117463	5.878349
O	5.993340	-3.307916	5.666417
H	6.945475	-3.279813	5.886738
O	5.901157	-4.703389	5.138388
H	2.907687	-6.227726	7.635476

MeHgSec
Nimag=0
E=-3.98367446

C	1.518038	-3.144879	7.372488
C	3.023351	-3.193151	7.080811
N	1.138452	-3.007073	8.795427
H	1.849858	-3.461827	9.377973
H	1.072009	-2.030364	9.076458
H	1.100174	-2.284726	6.827730
C	0.789814	-4.379929	6.772440
O	1.070339	-4.863697	5.690769
O	-0.195221	-4.857476	7.559736
H	-0.142607	-4.280896	8.378769
H	3.198448	-3.418256	6.029147
H	3.492185	-2.238162	7.331358
Se	4.024553	-4.540761	8.203898
Hg	3.139569	-6.695881	7.131514
C	2.410607	-8.511441	6.273916
H	1.495550	-8.260465	5.733525
H	3.173356	-8.896211	5.593861
H	2.204851	-9.230113	7.069632
H	5.519504	-4.751139	6.335980
O	5.253278	-6.113683	4.995415
H	4.714869	-5.744469	4.266863
O	5.953522	-4.874400	5.447591

Sec^-
Nimag=0
E= -3.33825608

C	1.447811	-3.060359	7.523798
C	2.696938	-3.302679	6.637491
N	1.707490	-2.988817	8.984399
H	2.509358	-3.633164	9.138230
H	2.076152	-2.064847	9.221122
H	0.987833	-2.107012	7.217192
C	0.327583	-4.107837	7.284962
O	-0.040860	-4.488322	6.188167
O	-0.268476	-4.504016	8.439467
H	0.272348	-3.996700	9.131264
H	2.378948	-3.743860	5.690495
H	3.181171	-2.341817	6.427129
Se	4.067288	-4.504585	7.496221
O	4.070407	-0.872191	8.986708
O	5.243992	-1.820340	8.895702
H	4.822313	-2.682800	8.519695
H	4.074186	-0.526878	8.073069

Tec
Nimag=0
E= -3.36332440

C	1.548249	-3.292151	7.152642
C	2.820874	-3.899254	6.555290
N	1.687531	-2.611102	8.456982
H	2.351673	-3.121218	9.047519
H	2.029897	-1.657667	8.348186
H	1.180360	-2.541250	6.435886
C	0.401540	-4.341789	7.234678
O	0.233864	-5.209404	6.404442
O	-0.411746	-4.167661	8.303094
H	0.001628	-3.413909	8.808215
H	2.606009	-4.424415	5.625066
H	3.590824	-3.144003	6.387452
Te	3.808786	-5.374247	7.872760
H	5.609465	-5.140625	5.927798
O	6.187804	-3.347721	6.301013
H	7.120158	-3.388813	6.592835
O	6.161800	-4.525412	5.381432
H	2.652101	-6.546529	7.555895

MeHgTec
Nimag=0
E= -3.95804519

C	1.590495	-3.197030	7.334849
C	3.096296	-3.416933	7.166874
N	1.140785	-2.706284	8.656949
H	1.718670	-3.127758	9.391909
H	1.224873	-1.693620	8.729897
H	1.279916	-2.447566	6.589307
C	0.770477	-4.464445	6.966899
O	1.074515	-5.226234	6.067104
O	-0.336031	-4.626444	7.720879
H	-0.283012	-3.881795	8.387886
H	3.327355	-3.831634	6.186290
H	3.641778	-2.480963	7.305510
Te	3.963872	-4.811451	8.670336
Hg	3.142736	-7.005495	7.263034
C	2.521042	-8.738733	6.155851
H	1.852528	-8.380547	5.370479
H	3.400306	-9.227162	5.730045
H	1.995404	-9.417404	6.830773
H	5.743263	-4.994543	6.783746
O	5.080688	-5.672600	5.105106
H	5.562141	-6.479956	4.836594
O	6.161027	-4.989025	5.880349

Cys (SAPE)
Nimag=0
E= -4.47342184

C	1.299248	-3.326130	7.376114
C	2.661630	-2.942149	6.764966
N	1.195166	-3.186318	8.843488
H	2.109151	-3.367284	9.269546
H	0.891013	-2.253698	9.116821
H	0.536272	-2.680456	6.917955
C	0.886238	-4.770346	6.973167
O	1.059659	-5.224821	5.856077
O	0.289990	-5.457610	7.963025
H	0.324449	-4.832346	8.744765
H	2.674801	-3.188407	5.701272
H	2.825924	-1.867132	6.882079
S	4.099312	-3.767333	7.592127
H	3.962462	-4.992569	6.936046
O	6.879386	-4.149655	5.084816

O	6.004271	-2.938703	5.148507
H	5.512273	-3.148989	5.994023
H	7.751246	-3.719832	4.988220
O	5.286515	-5.991801	3.667508
H	5.909759	-5.316991	4.021478
H	4.839134	-5.558653	2.921233
O	3.682397	-6.456220	5.942525
H	4.191897	-6.348473	5.100839
H	2.744540	-6.310601	5.705140

MeHgCys (SAPE)
Nimag=0
E= -5.04615475

C	-0.121239	0.862644	-0.414308
C	0.876176	0.871257	-1.589049
N	0.482041	1.013915	0.927924
H	1.436087	0.638363	0.909917
H	0.526908	1.989849	1.215230
H	-0.828355	1.689538	-0.570053
C	-1.000090	-0.420817	-0.439998
O	-1.482783	-0.878556	-1.457323
O	-1.190149	-0.966744	0.780908
H	-0.654157	-0.376674	1.388378
H	0.352740	0.612684	-2.510964
H	1.310004	1.870495	-1.695397
S	2.323886	-0.264877	-1.327367
Hg	1.368325	-2.484803	-1.693775
C	0.469801	-4.387100	-2.015498
H	0.782533	-4.761816	-2.992892
H	0.802984	-5.062106	-1.223727
H	-0.613422	-4.258645	-1.981114
O	3.632524	-0.378423	-4.202640
O	5.004719	0.159847	-3.917157
H	5.202785	0.538119	-4.795393
H	3.221540	-0.243052	-3.295935
O	6.090744	-2.145118	-2.725204
H	5.770338	-1.344060	-3.204687
H	6.709290	-1.809327	-2.054717
O	4.029799	-3.512360	-1.444889
H	4.747957	-3.024591	-1.925612
H	4.054048	-4.412523	-1.810159

Sec (SAPE)
Nimag=0
E= -4.43481239

C	0.959918	-3.373531	7.518622
C	2.085466	-2.721574	6.702040
N	1.113222	-3.304728	8.988946
H	2.104783	-3.386350	9.233331
H	0.759977	-2.426840	9.365803
H	0.020273	-2.861047	7.261417
C	0.717431	-4.856222	7.108300
O	0.747420	-5.249518	5.957420
O	0.415596	-5.658343	8.147989
H	0.489048	-5.058442	8.945560
H	1.938906	-2.882821	5.633586
H	2.134225	-1.650662	6.911998
Se	3.892404	-3.477371	7.156262
H	3.731003	-4.532205	6.074878
O	6.721507	-4.551625	5.409587
O	6.290478	-3.262174	4.790724

H	5.530617	-3.051542	5.397462
H	7.636331	-4.320624	5.663187
O	5.536957	-6.732668	4.008805
H	6.062331	-5.992491	4.389557
H	5.731433	-6.715342	3.056321
O	3.205935	-5.258844	4.511756
H	3.856249	-5.975618	4.319629
H	2.363366	-5.658148	4.803274

MeHgSec (SAPE)
Nimag=0
E= -5.02099710

C	-0.220752	0.915640	-0.376643
C	0.886722	1.031281	-1.434455
N	0.217776	1.045259	1.030655
H	1.184302	0.712488	1.115020
H	0.182680	2.011413	1.350702
H	-0.956048	1.709088	-0.576409
C	-1.020189	-0.408278	-0.545188
O	-1.361867	-0.848565	-1.625214
O	-1.317286	-1.010056	0.627336
H	-0.886862	-0.418251	1.311809
H	0.495134	0.770296	-2.417305
H	1.288498	2.047549	-1.457121
Se	2.490834	-0.130749	-1.044360
Hg	1.503893	-2.434409	-1.566994
C	0.601534	-4.319771	-2.007811
H	0.989407	-4.674411	-2.965415
H	0.855671	-5.019454	-1.208144
H	-0.477908	-4.167810	-2.059034
O	3.584265	-0.435	-4.138556
O	5.012991	0.012965	-4.046647
H	5.100652	0.426218	-4.927253
H	3.287290	-0.215222	-3.206322
O	6.087312	-2.391739	-3.044118
H	5.763874	-1.564731	-3.473593
H	6.865123	-2.121767	-2.527363
O	4.180874	-3.485251	-1.325293
H	4.840365	-3.097637	-1.956819
H	4.126747	-4.423977	-1.570966

PCox

Cys
Nimag=0
E= -3.49594477

C	1.525756	-3.459100	6.999856
C	1.968226	-4.423291	5.884882
N	2.474031	-2.447132	7.488379
H	3.442692	-2.782367	7.593393
H	2.491562	-1.622929	6.891274
H	0.654027	-2.917253	6.604450
C	0.959198	-4.263359	8.210863
O	0.400138	-5.335675	8.094679
O	1.127121	-3.626709	9.387283
H	1.667662	-2.817985	9.144587
H	1.115537	-5.027427	5.563449
H	2.408978	-3.898710	5.031340
S	3.285642	-5.634491	6.451395
H	5.305011	-3.948338	7.004983

O	5.337750	-3.184529	7.636210
H	5.867062	-3.486357	8.392714
O	4.629752	-5.175995	5.893741
H	2.824354	-6.596551	5.567722

MeHgCys
Nimag=0
E= -4.06967051

C	1.391184	-3.221925	7.246753
C	2.774860	-3.489247	6.640109
N	1.388872	-2.529375	8.556637
H	2.207030	-2.805799	9.104808
H	1.414395	-1.517276	8.445122
H	0.832559	-2.592795	6.539450
C	0.539205	-4.519775	7.357927
O	0.569215	-5.412158	6.531364
O	-0.261805	-4.545523	8.442391
H	-0.018976	-3.714225	8.943130
H	2.700947	-3.923097	5.641721
H	3.358418	-2.564271	6.590209
S	3.823210	-4.586198	7.759022
Hg	3.252791	-6.791175	6.638836
C	2.799949	-8.688405	5.746655
H	3.139134	-8.651246	4.709556
H	3.322985	-9.471193	6.299686
H	1.718676	-8.826491	5.801164
H	5.509301	-4.997132	5.843750
O	5.314714	-5.533442	5.018618
H	6.109314	-6.071335	4.867832
O	5.293323	-4.265319	7.379861

Cys^-
Nimag=0
E= -3.43503589

C	1.355026	-3.035891	7.306602
C	2.610432	-3.258265	6.441425
N	1.539541	-2.310648	8.586837
H	2.495938	-2.378074	8.974924
H	1.342439	-1.318228	8.472498
H	0.621618	-2.466318	6.707331
C	0.621675	-4.376803	7.605119
O	0.379110	-5.227292	6.766079
O	0.205306	-4.464593	8.890308
H	0.571560	-3.598269	9.277458
H	2.332313	-3.893074	5.591177
H	2.989498	-2.298429	6.065630
S	4.052914	-4.062573	7.294890
O	4.138498	-1.951438	10.012404
O	4.979007	-2.824039	7.805898
H	4.550638	-2.343589	9.095280
H	4.339946	-1.002611	9.960365

Sec
Nimag=0
E= -3.44936926

C	1.479578	-3.506822	6.952849
C	2.077283	-4.530873	5.979538
N	2.313503	-2.367101	7.370558
H	3.311006	-2.606490	7.482537
H	2.255255	-1.598345	6.705519
H	0.585976	-3.091913	6.462463
C	0.920562	-4.221262	8.221758
O	0.486960	-5.357722	8.208696
O	0.957306	-3.441653	9.318165
H	1.449975	-2.623479	9.004787
H	1.303564	-5.183918	5.572934
H	2.662232	-4.068655	5.180894
Se	3.422176	-5.783769	6.888327
H	5.282114	-3.846659	6.974843
O	5.196123	-2.968217	7.444883
H	5.821837	-2.999717	8.186986
O	4.916660	-5.359740	6.202854
H	2.968491	-6.935567	6.002208

MeHgSec
Nimag=0
E= -4.03298332

C	1.372711	-3.204897	7.239370
C	2.773746	-3.474786	6.692570
N	1.311470	-2.442962	8.510506
H	2.123534	-2.652236	9.095590
H	1.302611	-1.438260	8.343458
H	0.820943	-2.624272	6.485069
C	0.543839	-4.514291	7.392144
O	0.614237	-5.445094	6.612338
O	-0.282033	-4.506894	8.458937
H	-0.073448	-3.644922	8.921535
H	2.749452	-3.967003	5.720962
H	3.367778	-2.559330	6.631544
Se	3.904660	-4.638371	7.950824
Hg	3.300064	-6.881869	6.671415
C	2.811051	-8.717061	5.664877
H	3.141927	-8.615332	4.629609
H	3.329032	-9.538877	6.162925
H	1.728523	-8.841917	5.722763
H	5.523315	-5.037334	5.888413
O	5.271278	-5.571712	5.066568
H	6.052703	-6.109604	4.858016
O	5.491907	-4.290378	7.385170

Sec^-
Nimag=0
E= -3.40393795

C	1.331671	-3.025053	7.321553
C	2.590821	-3.225760	6.469767
N	1.508299	-2.337937	8.624585
H	2.483219	-2.326900	8.983344
H	1.211069	-1.366199	8.563467
H	0.605695	-2.438759	6.729626
C	0.595453	-4.375728	7.574728
O	0.342181	-5.190203	6.702710
O	0.200763	-4.513022	8.859753
H	0.585485	-3.662694	9.273106
H	2.326176	-3.756314	5.549939
H	3.061023	-2.266813	6.227306
Se	4.080974	-4.269535	7.369677
O	4.108475	-1.844998	9.892838
O	5.161713	-2.949161	7.913212
H	4.611810	-2.352501	9.053708
H	4.320631	-0.908556	9.744907

Tec
Nimag=0
E= -3.43099731

C	1.446570	-3.510986	6.977210
C	2.061584	-4.538187	6.015866
N	2.250568	-2.329966	7.338937
H	3.254119	-2.546330	7.442593
H	2.165003	-1.594922	6.639440
H	0.525339	-3.138811	6.503112
C	0.946619	-4.193330	8.285014
O	0.591370	-5.357016	8.338141
O	0.944057	-3.360021	9.340958
H	1.396767	-2.535574	8.984326
H	1.294532	-5.186938	5.588257
H	2.650581	-4.067718	5.224537
Te	3.503434	-5.942117	6.974440
H	5.300195	-3.830724	6.936762
O	5.123366	-2.961532	7.411758
H	5.802580	-2.887483	8.101729
O	5.111286	-5.363557	6.217053
H	3.042632	-7.146689	5.840666

MeHgTec
Nimag=0
E= -4.01629138

C	1.360204	-3.182852	7.245995
C	2.786553	-3.433130	6.761093
N	1.229283	-2.379198	8.487101
H	2.030324	-2.530506	9.104211
H	1.184678	-1.382341	8.282932
H	0.819462	-2.641179	6.454853
C	0.559941	-4.507676	7.412885
O	0.676485	-5.458831	6.664119
O	-0.295293	-4.493078	8.457403
H	-0.126259	-3.610461	8.896630
H	2.807765	-3.956912	5.805566
H	3.356300	-2.503473	6.683489
Te	4.048655	-4.672629	8.145390
Hg	3.331577	-6.981937	6.717082
C	2.780660	-8.747365	5.608199
H	3.099478	-8.592401	4.575728
H	3.283686	-9.610593	6.048331
H	1.696047	-8.848518	5.674802
H	5.580271	-5.086543	5.871148
O	5.246774	-5.616206	5.072206
H	6.000419	-6.159771	4.789390
O	5.722291	-4.321800	7.346046

Cys (SAPE)
Nimag=0
E= -4.56192625

C	1.364519	-3.200531	7.503178
C	2.030487	-3.945998	6.335524
N	2.288199	-2.689163	8.543707
H	3.168985	-3.208623	8.535170
H	2.501069	-1.703398	8.406351
H	0.810764	-2.349111	7.082538
C	0.272946	-4.098705	8.162301
O	-0.522352	-4.749441	7.515972
O	0.280374	-4.044584	9.510702
H	1.060980	-3.451810	9.718126
H	1.256867	-4.361761	5.679361
H	2.676694	-3.281867	5.752253
S	3.011365	-5.412778	6.852231
H	3.503223	-6.139544	4.786878
O	6.077580	-3.778189	5.751274
O	4.359534	-4.712401	7.581458
H	5.001729	-4.417790	6.862172
H	6.993424	-3.839513	6.070882
O	5.965078	-4.979810	3.332376
H	6.080724	-4.195999	4.840767
H	5.828799	-4.403827	2.561595
O	3.683206	-6.369458	3.835673
H	5.139118	-5.533149	3.410422
H	3.695428	-7.341474	3.801184

MeHgCys (SAPE)
Nimag=0
E= -5.11530121

C	-0.063478	0.941590	-1.071505
C	1.209704	0.270146	-1.596486
N	0.139601	2.044281	-0.104589
H	0.911625	1.820301	0.528176
H	0.385238	2.909385	-0.584452
H	-0.592369	1.363508	-1.938324
C	-1.067234	-0.075420	-0.460710
O	-1.186095	-1.223798	-0.851907
O	-1.821899	0.443101	0.527054
H	-1.442183	1.358235	0.667700
H	1.004308	-0.388434	-2.441124
H	1.947200	1.022620	-1.898157
S	2.075521	-0.663417	-0.211676
Hg	1.217084	-2.951911	-0.830659
C	0.524919	-4.911484	-1.342784
H	0.862117	-5.127983	-2.358317
H	0.945398	-5.626243	-0.632706
H	-0.564989	-4.896510	-1.285216
O	3.603936	-0.644150	-0.554816
O	4.053302	1.736477	-2.234009
H	4.154271	1.036026	-1.555579
H	3.833063	-1.649054	-1.807416
O	3.372242	-0.125870	-4.376469
H	3.928374	1.220658	-3.061783
H	3.900385	-0.179035	-5.189967
O	3.726019	-2.255879	-2.615109
H	3.563016	-0.962236	-3.879693
H	4.502396	-2.839610	-2.617080

Sec (SAPE)
Nimag=0
E= -4.53432980

C	1.348222	-3.185891	7.498233
C	1.941233	-3.976459	6.328331
N	2.326987	-2.688394	8.493995
H	3.186763	-3.244826	8.470821
H	2.575242	-1.716262	8.322871
H	0.811512	-2.321362	7.079976
C	0.247586	-4.025260	8.217518
O	-0.586730	-4.671320	7.616541
O	0.299074	-3.928017	9.561828
H	1.109400	-3.359962	9.724341
H	1.145771	-4.367986	5.686828

H	2.635684	-3.375268	5.736554
Se	2.935566	-5.610121	6.917345
H	3.538875	-6.242586	4.694890
O	6.038484	-3.763208	5.789983
O	4.399964	-4.793676	7.684732
H	4.999038	-4.487170	6.941053
H	6.959072	-3.779914	6.101093
O	5.976404	-4.902664	3.326035
H	6.058633	-4.158162	4.871755
H	5.823076	-4.305052	2.575182
O	3.748798	-6.423992	3.740863
H	5.173868	-5.490503	3.375205
H	3.836218	-7.390872	3.676124

MeHgSec (SAPE)
Nimag=0
E= -5.08108090

C	-0.090328	1.016307	-1.044957
C	1.205776	0.377976	-1.537324
N	0.056412	2.159862	-0.113703
H	0.835997	1.999990	0.528818
H	0.260186	3.019541	-0.621990
H	-0.628254	1.391283	-1.928916
C	-1.064502	-0.017686	-0.412127
O	-1.132595	-1.182097	-0.763827
O	-1.849794	0.504210	0.550951
H	-1.507748	1.437465	0.664386
H	1.042347	-0.310085	-2.365323
H	1.951129	1.125854	-1.825356
Se	2.143903	-0.634763	-0.024265
Hg	1.303095	-2.983021	-0.870166
C	0.620753	-4.890942	-1.571211
H	0.961732	-5.000682	-2.602300
H	1.049425	-5.666112	-0.933243
H	-0.468913	-4.890492	-1.512399
O	3.811822	-0.604860	-0.509402
O	4.082376	1.625639	-2.334716
H	4.190001	0.947560	-1.629660
H	3.944889	-1.660683	-1.675320
O	3.132474	-0.371365	-4.285212
H	3.867568	1.081586	-3.123430
H	3.535709	-0.472598	-5.163052
O	3.812154	-2.326091	-2.447594
H	3.446044	-1.150878	-3.756785
H	4.609814	-2.879622	-2.469476

TSox

Cys
Nimag=-605.28
E= -3.39122150

C	1.252820	-3.335887	7.332871
C	2.619413	-3.546618	6.663296
N	1.238868	-2.551453	8.583172
H	2.030047	-2.798870	9.181285
H	1.280431	-1.549442	8.403525
H	0.626038	-2.797704	6.606679
C	0.520276	-4.689958	7.563617
O	0.678156	-5.658560	6.850423
O	-0.325629	-4.672402	8.617241
H	-0.184731	-3.784763	9.044483
H	2.524216	-4.050783	5.700214
H	3.138119	-2.595887	6.516229
S	3.785709	-4.554624	7.669062
H	6.067220	-4.457305	6.542667
O	6.889420	-4.368620	5.149547
H	6.860152	-5.275880	4.789231
O	5.139688	-4.578683	6.189083
H	3.182626	-5.742594	7.414452

MeHgCys
Nimag=-517.19
E=-3.97424355

C	1.249326	-3.318926	7.289373
C	2.607204	-3.572890	6.619726
N	1.293612	-2.716468	8.640381
H	2.114314	-3.050802	9.151369
H	1.342211	-1.699857	8.598472
H	0.690819	-2.631004	6.637941
C	0.379673	-4.606511	7.338547
O	0.374252	-5.446181	6.456009
O	-0.388378	-4.695663	8.440963
H	-0.123261	-3.899887	8.986900
H	2.478477	-3.989401	5.620193
H	3.173060	-2.640821	6.536197
S	3.700137	-4.708058	7.589743
Hg	2.921828	-6.911297	6.827245
C	2.350227	-8.832793	6.130204
H	2.935234	-9.058623	5.236710
H	2.547680	-9.562896	6.917345
H	1.284437	-8.782794	5.900457
H	5.932873	-4.296654	6.435055
O	6.796981	-4.331455	4.997249
H	7.029106	-5.273314	4.887850
O	5.114675	-4.752552	6.107487

Cys^-
Nimag=-218.08
E= -3.35195480

C	1.279118	-3.132053	7.456023
C	2.626071	-3.263515	6.684613
N	1.434873	-3.025584	8.930056
H	2.333012	-3.509403	9.131111
H	1.553911	-2.050911	9.207270
H	0.759642	-2.233492	7.091258
C	0.301463	-4.296053	7.149361
O	0.020392	-4.677249	6.027735
O	-0.273440	-4.814298	8.266645
H	0.162139	-4.267244	8.995850
H	2.410285	-3.683457	5.697215
H	3.051090	-2.261548	6.557231
S	3.883515	-4.291452	7.556533
O	5.178558	-0.626276	9.426573
O	5.019052	-2.011730	8.418658
H	4.495179	-2.581938	9.017197
H	4.714198	-0.048931	8.793596

Sec
Nimag=-548.24
E= -3.36315447

C	1.245364	-3.334047	7.319799

C	2.614903	-3.583823	6.685914
N	1.219826	-2.521768	8.553209
H	1.998512	-2.765238	9.169424
H	1.277451	-1.524580	8.352311
H	0.645810	-2.796473	6.569352
C	0.470874	-4.664402	7.556727
O	0.596488	-5.639459	6.846921
O	-0.375021	-4.614670	8.610255
H	-0.214520	-3.724163	9.025067
H	2.543626	-4.146535	5.755249
H	3.164411	-2.656423	6.513533
Se	3.859335	-4.649940	7.833495
H	6.176011	-4.605922	6.535497
O	6.946875	-4.277339	5.139457
H	6.761777	-5.054168	4.577132
O	5.225815	-4.517107	6.238863
H	3.165300	-5.933977	7.534870

MeHgSec
E= -3.95467177
Nimg=-456.15

C	1.235868	-3.304822	7.296914
C	2.619186	-3.515564	6.679009
N	1.207181	-2.686867	8.642205
H	2.016717	-2.989259	9.190024
H	1.225652	-1.669687	8.590403
H	0.675787	-2.641512	6.620242
C	0.404165	-4.618014	7.326444
O	0.452114	-5.462210	6.449868
O	-0.398064	-4.723442	8.403109
H	-0.178538	-3.914913	8.950318
H	2.558007	-3.968624	5.690452
H	3.168204	-2.573968	6.608739
Se	3.825031	-4.703044	7.768038
Hg	2.953530	-6.965572	6.901049
C	2.331325	-8.849751	6.124411
H	2.928573	-9.062790	5.235980
H	2.487439	-9.611387	6.890549
H	1.273293	-8.751879	5.875427
H	6.020064	-4.271797	6.358104
O	6.772069	-4.383009	4.841335
H	7.020937	-5.324882	4.778342
O	5.205944	-4.785854	6.124455

Sec^-
Nimag=-196.01
E= -3.32997829

C	1.264401	-3.138328	7.438985
C	2.590017	-3.261253	6.653645
N	1.423116	-2.998101	8.910850
H	2.309454	-3.489559	9.137223
H	1.555006	-2.018412	9.163851
H	0.724334	-2.251980	7.069859
C	0.292715	-4.316372	7.159846
O	0.022499	-4.732540	6.048610
O	-0.295731	-4.795534	8.287140
H	0.135844	-4.231384	9.004403
H	2.380801	-3.671049	5.663233
H	3.048313	-2.273523	6.554755
Se	3.961124	-4.413622	7.564784
O	5.159955	-0.615233	9.483515
O	5.104381	-1.963144	8.443965
H	4.554532	-2.576690	8.979355
H	4.718298	-0.028410	8.842907

Tec
Nimag=-371.66
E= -3.34373240

C	1.265078	-3.350550	7.288135
C	2.625840	-3.716798	6.698953
N	1.264599	-2.472287	8.476851
H	2.008122	-2.735274	9.128076
H	1.401095	-1.494519	8.224608
H	0.712186	-2.814859	6.500648
C	0.399297	-4.612702	7.573366
O	0.466478	-5.625993	6.911426
O	-0.458139	-4.455969	8.608681
H	-0.248128	-3.556708	8.979880
H	233242	-4.343892	5.811952
H	3.232343	-2.839068	6.467866
Te	3.930587	-4.883207	8.029510
H	6.64059	-4.729331	6.601997
O	6.886182	-3.984696	5.117290
H	6.569695	-4.574196	4.405731
O	5.336416	-4.502457	6.357040
H	3.133885	-6.317529	7.635065

MeHgTec
E= -3.93937587
Nimg=-317.70

C	1.273513	-3.310041	7.271697
C	2.695093	-3.496374	6.745898
N	1.141615	-2.669694	8.600999
H	1.916621	-2.947602	9.209113
H	1.148417	-1.653397	8.531223
H	0.739955	-2.669935	6.551715
C	0.466754	-4.638406	7.270530
O	0.583901	-5.494767	6.412250
O	-0.400541	-4.743786	8.296141
H	-0.233499	-3.921218	8.841109
H	2.712304	-3.970691	5.765389
H	3.233065	-2.547790	6.693623
Te	3.997635	-4.772220	7.997770
Hg	2.962792	-7.119991	7.016704
C	2.224136	-8.950868	6.180153
H	2.844156	-9.206622	5.318858
H	2.277431	-9.729556	6.943384
H	1.191862	-8.760049	5.881556
H	6.242627	-4.352403	6.492552
O	6.723823	-4.141570	4.746529
H	6.712074	-5.048327	4.385132
O	5.350748	-4.633541	6.193091

Cys (SAPE)
Nimag=-189.48
E= -4.45331012

C	1.197481	-3.246004	7.434855
C	2.477145	-3.275855	6.582245
N	1.362407	-2.807871	8.837717
H	2.242684	-3.155676	9.224148
H	1.361290	-1.792522	8.919972

H	0.503376	-2.542763	6.952602
C	0.460160	-4.618297	7.398701
O	0.411928	-5.318626	6.411536
O	-0.155304	-4.930152	8.564495
H	0.090606	-4.190484	9.184295
H	2.258104	-3.622837	5.570903
H	2.933827	-2.283598	6.540702
S	3.751933	-4.410536	7.285907
H	3.583148	-5.448336	6.284907
O	6.751542	-3.566417	4.683280
O	5.305683	-3.851061	5.951100
H	5.978633	-3.593156	6.611344
H	6.255320	-2.887814	4.188612
O	5.973898	-5.980951	3.968329
H	6.303678	-5.036436	4.192875
H	6.086881	-6.085293	3.009824
O	3.561336	-6.224822	4.973422
H	4.497274	-6.177274	4.563001
H	3.335709	-7.162146	5.097324

MeHgCys (SAPE)
Nimag=-198.12
E= -5.03447410

C	-0.352957	0.801984	-0.763836
C	1.035309	0.483108	-1.334786
N	-0.371250	1.609803	0.476454
H	0.435727	1.376879	1.060493
H	-0.331176	2.607307	0.273713
H	-0.897046	1.366261	-1.535293
C	-1.202897	-0.480245	-0.543926
O	-1.160167	-1.449016	-1.280750
O	-2.016028	-0.408743	0.528138
H	-1.782634	0.468238	0.950364
H	0.952671	-0.077994	-2.266665
H	1.594022	1.401953	-1.530343
S	2.106023	-0.457107	-0.149528
Hg	1.441475	-2.766485	-0.740721
C	0.878512	-4.769471	-1.177967
H	1.188944	-4.977737	-2.203581
H	1.370526	-5.444820	-0.475627
H	-0.206938	-4.828156	-1.080800
O	3.799489	-0.004416	-1.264219
O	5.167451	0.640143	-2.533138
H	5.888257	0.082742	-2.186145
H	3.714091	-0.792329	-1.855693
O	3.807731	-0.190573	-4.558053
H	4.439183	0.173994	-3.818367
H	4.328510	-0.236107	-5.375706
O	3.130744	-2.238717	-3.056534
H	3.353673	-1.551454	-3.769580
H	3.782828	-2.949134	-3.173161

Sec (SAPE)
Nimag= -93.28
E=-4.42402826

C	1.103452	-3.161361	7.429602
C	2.443134	-2.937904	6.715922
N	1.092910	-2.882054	8.883610
H	1.987778	-3.151619	9.300421
H	0.937107	-1.894297	9.077765
H	0.365056	-2.491373	6.963922
C	0.546240	-4.594999	7.175009
O	0.627790	-5.161784	6.106582
O	-0.077724	-5.128291	8.251900
H	0.037270	-4.443604	8.968117
H	2.380630	-3.213635	5.662946
H	2.768128	-1.900093	6.816567
Se	3.909692	-4.071794	7.484805
H	3.664589	-5.156791	6.408663
O	6.762234	-3.624659	4.408611
O	5.572585	-3.656911	5.714910
H	6.188015	-3.230994	6.344991
H	6.218292	-3.058492	3.827861
O	6.000113	-6.241726	4.122008
H	6.332020	-5.297542	4.180252
H	6.194912	-6.532186	3.216038
O	3.460840	-5.857400	4.954497
H	4.363091	-6.135839	4.618077
H	2.882858	-6.637195	5.002832

MeHgSec (SAPE)
Nimag=-93.28
E=-5.01517281

C	-0.437985	1.037946	-0.766456
C	0.969246	0.763382	-1.298005
N	-0.518777	1.821514	0.487848
H	0.274095	1.596522	1.094477
H	-0.497023	2.823471	0.305213
H	-0.973151	1.606016	-1.542734
C	-1.267068	-0.266451	-0.602152
O	-1.188811	-1.212391	-1.364553
O	-2.107021	-0.240714	0.452046
H	-1.906068	0.631590	0.899606
H	0.940903	0.186476	-2.221578
H	1.521368	1.689086	-1.470554
Se	2.138777	-0.238337	-0.000559
Hg	1.368643	-2.603329	-0.707398
C	0.729560	-4.566985	-1.245307
H	1.164368	-4.791896	-2.220852
H	1.071567	-5.277114	-0.490036
H	-0.360368	-4.545232	-1.296918
O	3.838564	0.249671	-1.381226
O	5.062869	0.873704	-2.714712
H	5.849363	0.491508	-2.283212
H	3.780253	-0.621405	-1.848218
O	3.913262	-0.401387	-4.674664
H	4.449828	0.133802	-3.981406
H	4.486418	-0.506363	-5.450789
O	3.361536	-2.251443	-2.861226
H	3.547081	-1.679156	-3.672188
H	4.070760	-2.915031	-2.846280

TSiso

Cys
Nimag=-1462.67
E=-2.91136466

C	1.251550	-3.388189	7.211226

C	2.402183	-3.919473	6.338853
N	1.667898	-2.689806	8.448474
H	2.620597	-2.958582	8.720523
H	1.630908	-1.678193	8.346427
H	0.664336	-2.698528	6.589588
C	0.262574	-4.546120	7.562855
O	-0.113124	-5.358423	6.742563
O	-0.139283	-4.524967	8.848254
H	0.378609	-3.761193	9.243927
H	1.999750	-4.492253	5.496099
H	3.041527	-3.106522	5.984159
S	3.460018	-5.118553	7.275339
O	4.258448	-4.153355	8.437314
H	4.743075	-4.536898	7.109398

Sec
Nimag=-1304.37
E=-2.87701023

C	1.226359	-3.387839	7.221488
C	2.326995	-3.882147	6.274084
N	1.694505	-2.745515	8.468956
H	2.633888	-3.084894	8.724473
H	1.722973	-1.731622	8.387069
H	0.612456	-2.668151	6.660409
C	0.244941	-4.552444	7.571006
O	-0.133136	-5.364185	6.750101
O	-0.143349	-4.541154	8.860087
H	0.387986	-3.784461	9.256062
H	1.899213	-4.397572	5.409929
H	2.994659	-3.075727	5.966756
Se	3.482097	-5.269600	7.188885
O	4.231388	-4.254662	8.549894
H	4.810370	-4.520782	7.095621

Tec
Nimag=-1242.70
E=-2.85575642

C	1.168629	-3.389946	7.252263
C	2.175011	-3.895749	6.201985
N	1.675024	-2.485065	8.296398
H	2.506730	-2.910583	8.727607
H	1.898625	-1.562011	7.929272
H	0.379075	-2.852985	6.702422
C	0.422837	-4.563734	7.943441
O	0.321745	-5.671220	7.449989
O	-0.142145	-4.219651	9.117903
H	0.235867	-3.320243	9.321092
H	1.670968	-4.430207	5.392729
H	2.775092	-3.076338	5.802437
Te	3.555711	-5.378791	7.101982
O	3.523233	-4.653127	8.968485
H	4.632149	-4.249508	7.829536

Fictitious reactant
Nimag=0
E=-1.68539693

O	6.759881	-4.568380	5.365621
O	5.644527	-3.802030	6.001700
H	4.873919	-4.092005	5.430063
H	7.203544	-3.846864	4.877286
O	5.516856	-6.702663	3.980597
H	6.122109	-6.053972	4.414442
H	5.903080	-6.887466	3.108145
O	3.617336	-4.759626	4.378769
H	4.110837	-5.579552	4.116067
H	2.832411	-5.066943	4.863110

Table S2. Activation energies (kcal mol^{-1}) relative to RC for Cys, Sec, MeHgCys and MeHgSec (SAPE mechanism) in gas phase. Level of theory ZORA-B3LYP-D3(BJ)/TZ2P // ZORA-B3LYP/TZ2P.

	ΔE
Cys	22.85
Sec	16.50
MeHgCys	18.51
MeHgSec	14.65

Table S3. Energies (kcal mol^{-1}) relative to free reactants for the minimal oxidation mechanism of (MeHg)X, where (X=Cys, Sec), and anionic mechanism (Cys^-, Sec^-) in water. Level of theory COSMO-ZORA-BLYP-D3(BJ)/TZ2P // ZORA-BLYP-D3(BJ)/TZ2P.

	RC_{ox}	TS_{ox}	PC_{ox}	P_{ox}	TS_{iso}	P_{iso}
Cys	-3.95	6.74	-48.43	-44.21	-3.70	-52.28
Sec	-3.87	1.97	-41.88	-36.48	-5.1	-57.88
Cys^-	-8.51	-3.36	-58.72	-49.91	-	-
Sec^-	-7.96	-3.70	-50.33	-44.25	-	-
MeHgCys	-6.40	2.03	-48.44	-41.22	-	-
MeHgSec	-6.09	-2.22	-43.11	-34.83	-	-

Table S4. Energies (kcal mol^{-1}) relative to free reactants for the SAPE RC and TS of Cys, Sec, MeHgCys and MeHgSec in water. Level of theory COSMO-ZORA-BLYP-D3(BJ)/TZ2P // ZORA-BLYP-D3(BJ)/TZ2P.

	RC	TS
Cys	-11.48	-6.14
Sec	-10.86	-5.93
MeHgCys	-14.54	-12.41
MeHgSec	-14.27	-14.77[a]

[a]The TS is at slightly lower energy with respect to the relative RC. This is because data are single point energy values.

Table S5. Gibbs free energies (kcal mol^{-1}) relative to free reactants for the minimal and anionic mechanisms, gas phase. Level of theory ZORA-BLYP-D3(BJ)/TZ2P.

	RCox	TSox	PCox	Pox	TSiso	Piso
Cys	3.55	26.00	-36.34	-37.83	-0.15	-48.19
Sec	3.50	22.08	-28.98	-29.26	-0.02	-53.39
Tec	3.39	14.4	-37.96	-36.89	-6.12	-63.27
Cys^-	-9.93	-2.99	-54.98	-49.79	-	
Sec^-	-9.08	-3.7	-50.33	-44.25	-	
MeHgCys	2.74	21.00	-36.33	-34.09	-	-
MeHgSec	3.04	17.64	-29.11	-25.26	-	-
MeHgTec	4.26	13.05	-34.43	-29.79	-	-

Table S6. Gibbs free energies (kcal mol^{-1}) relative to free reactants for the SAPE oxidation mechanism of (MeHg)X, where (X=Cys, Sec) in gas phase. Level of theory ZORA-BLYP-D3(BJ)/TZ2P.

	RC	TS	PC	P
Cys	8.9	20.17	-45.53	-48.19
Sec	10.81	17.52	-49.50	-53.39
MeHgCys	9.09	16.97	-32.67	-34.09
MeHgSec	9.29	13.81	-26.78	-25.26

Table S7. ASA-EDA for the stationary points for Cys and MeHgCys oxidation on both mechanisms studied (minimal and SAPE). Energies (kcal mol^{-1}) relative to Cys/MeHgCys and H_2O_2 (minimal) and fictitious reactant (SAPE mechanism). Level of theory ZORA-BLYP-D3(BJ)/TZ2P

			ΔE	ΔE_{strain}	ΔE_{int}	ΔE_{Pauli}	ΔE_{elstat}	ΔE_{OI}	ΔE_{disp}
Cys	minimal	RC	-6.01	0.2	-6.21	10.26	-8.01	-5.77	-2.69
		TS	17.84	43.54	-25.70	126.61	-57.96	-91.83	-2.52
	SAPE	RC	-4.49	8.89	-13.38	32.66	-23.04	-17.85	-5.15
		TS	5.77	41.29	-35.52	142.42	-67.33	-105.59	-5.02
MeHgCys	minimal	RC	-8.61	0.29	-8.90	17.77	-12.98	-9.38	-4.31
		TS	12.79	42.84	-30.05	117.90	-55.20	-89.34	-3.41
	SAPE	RC	-5.44	9.02	-14.46	27.05	-20.87	-14.95	-5.69
		TS	1.89	34.41	-32.52	120.02	-58.12	-87.82	-6.60

Table S8. ASA-EDA for the stationary points for Sec and MeHgSec oxidation on both mechanisms studied (minimal and SAPE). Energies (kcal mol^{-1}) relative to Cys/MeHgCys and H_2O_2 (minimal) and fictitious reactant (SAPE mechanism). Level of theory ZORA-BLYP-D3(BJ)/TZ2P

			ΔE	ΔE_{strain}	ΔE_{int}	ΔE_{Pauli}	ΔE_{elstat}	ΔE_{OI}	ΔE_{disp}
Sec	stepwise	RC	-6.02	0.21	-6.23	9.90	-7.61	-5.63	-2.89
		TS	14.06	43.99	-29.93	117.59	-55.68	-89.19	-2.65
	SAPE	RC	-4.00	9.06	-13.06	36.68	-23.73	-20.54	-5.47
		TS	2.76	24.84	-22.08	80.18	-41.30	-55.68	-5.28
MeHgSec	minimal	RC	-8.44	0.31	-8.75	16.82	-12.22	-8.70	-4.65
		TS	9.76	42.63	-32.87	109.93	-52.87	-86.45	-3.48
	SAPE	RC	-4.96	8.83	-13.79	25.16	-19.01	-14.02	-5.92
		TS	-1.30	28.01	-29.31	93.72	-47.38	-69.24	-6.41

Table S9. ASA-EDA for the stationary points for Tec and MeHgTec minimal oxidation. Energies (kcal mol^{-1}) relative to Tec/MeHgTec and H_2O_2 fragments. Level of theory ZORA-BLYP-D3(BJ)/TZ2P

		ΔE	ΔE_{strain}	ΔE_{int}	ΔE_{Pauli}	ΔE_{elstat}	ΔE_{OI}	ΔE_{disp}
Tec	RC	-6.04	0.19	-6.23	9.90	-7.49	-5.43	-3.21
	TS	6.25	39.59	-33.34	125.73	-62.26	-93.82	-2.99
MeHgTec	RC	-7.82	0.38	-8.20	14.52	-10.00	-7.84	-4.88
	TS	3.89	38.51	-34.62	115.46	-57.40	-89.07	-3.61

Table S10. ASA comparison for Cys, Sec, MeHgCys, MeHgSec (SAPE mechanism) employing as reference state the free reactants or the fictitious reactant. Energies in kcal mol^{-1}. Level of theory ZORA-BLYP-D3(BJ)/TZ2P.

		Free reactants			Fictitious reactant		
		ΔE	ΔE_{strain}	ΔE_{int}	ΔE	ΔE_{strain}	ΔE_{int}
Cys	RC	-22.92	-9.54	-13.38	-4.49	8.89	-13.38
	TS	-12.65	22.85	-35.52	5.77	41.29	-35.52
MeHgCys	RC	-23.87	-9.41	-14.46	-5.44	9.02	-14.46
	TS	-16.53	15.99	-32.52	1.89	34.41	-32.52
Sec	RC	-22.43	-9.37	-13.06	-4.00	9.06	-13.06
	TS	-15.67	6.41	-22.08	2.76	24.84	-22.08
MeHgSec	RC	-23.29	-9.50	-13.79	-4.96	8.83	-13.79
	TS	-19.73	9.28	-29.01	-1.30	28.01	-29.31

Table S11. EDA for Cys^- and Sec^- (anionic mechanism) employing as reference state the free reactants. Energies in kcal mol^{-1}. Level of theory: ZORA-BLYP-D3(BJ)/TZ2P.

	ΔE_{Pauli}	ΔE_{elstat}	ΔE_{OI}	ΔE_{disp}
Cys^-	1.57	6.1	-8.04	0.95
Sec^-	2.88	4	-8.36	0.96

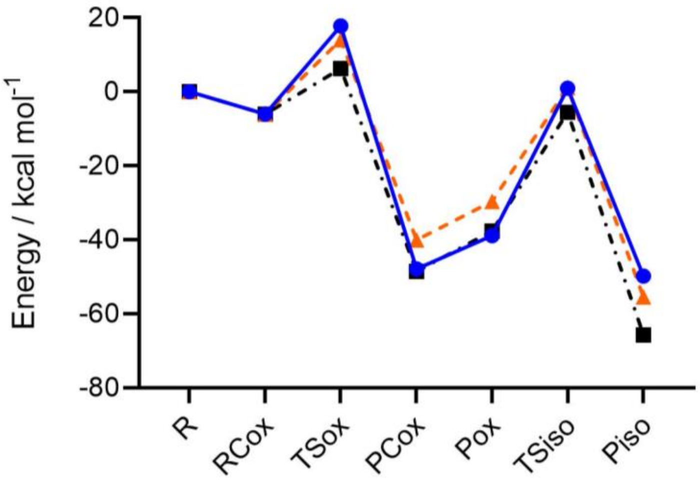

Figure S1. Stationary points for the stepwise oxidation of Cys (blue, solid), Sec (orange, dashed) and Tec (black, dashed-dotted). Level of theory ZORA-BLYP-D3(BJ)/TZ2P.

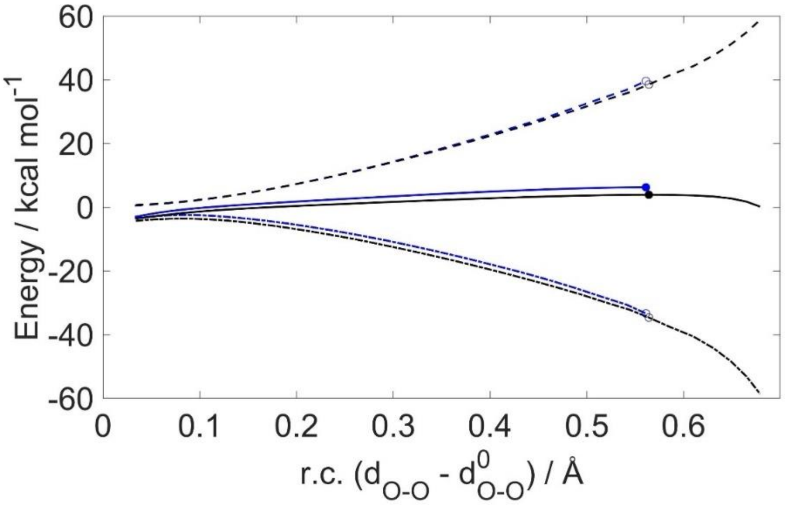

Figure S2. ASA along the r.c. for the oxidation of Tec (blue) and MeHgTec (black). Solid lines represent IRC profiles, dashed lines represent $\Delta E_{strain,}$ while dashed-dotted lines represent $\Delta E_{int.}$ $d^0_{O\text{-}O}$ refers to the O-O bond length in the RC. Level of theory ZORA-BLYP-D3(BJ)/TZ2P

Figure S3. Molecular orbitals of (MeHg)Cys and of H_2O_2 in the geometry they possess at the transition states of the respective oxidation reactions, mainly responsible for ΔE_{oi}. Isosurface value 0.05 a.u. Level of theory: ZORA-BLYP-D3(BJ)/TZ2P. *Top* Cys transition state geometry. Left: Cys HOMO; right: H_2O_2 LUMO. *Bottom*: MeHgCys transition state geometry. Left: MeHgCys HOMO; right: H_2O_2 LUMO.

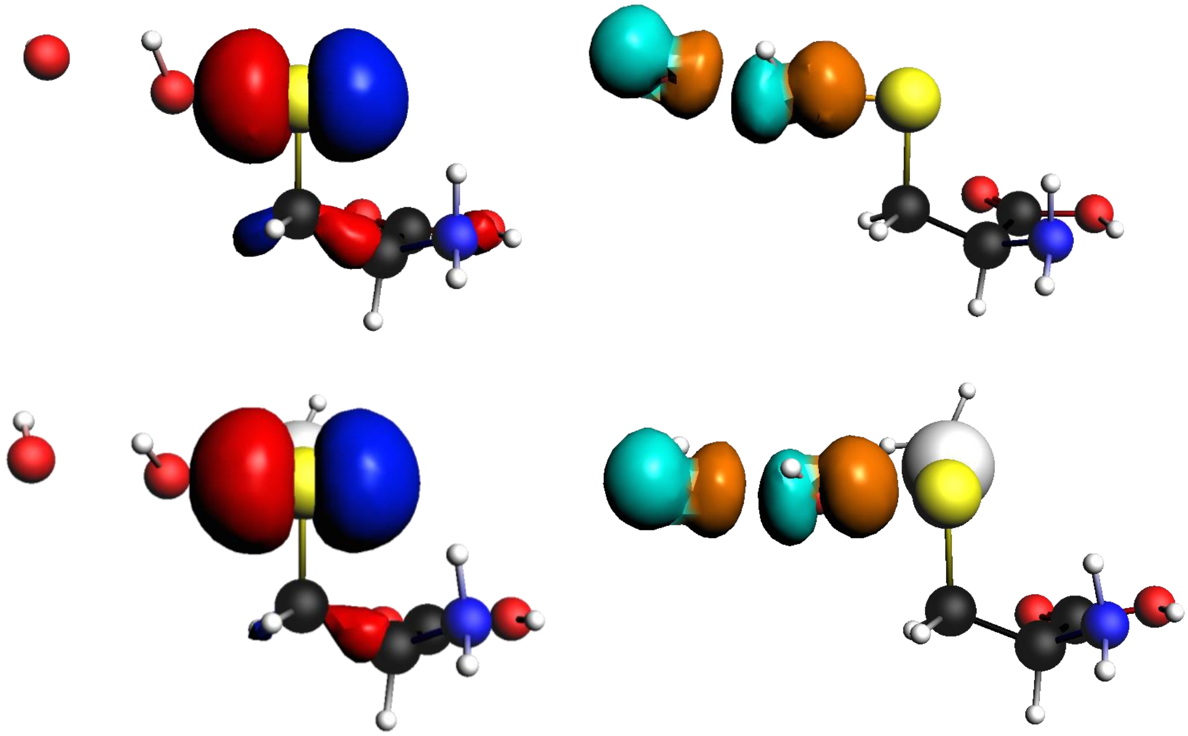

Supplement: Supplementary file 1 — ic0c03619_si_001.pdf [file ic0c03619_si_001.pdf]
